# From Membrane Composition to Antimicrobial Strategies: Experimental and Computational Approaches to AMP Design and Selectivity

**DOI:** 10.1002/smll.202411476

**Published:** 2025-06-17

**Authors:** Paolo Rossetti, Marius F.W. Trollmann, Christina Wichmann, Thomas Gutsmann, Christian Eggeling, Rainer A. Böckmann

**Affiliations:** ^1^ Computational Biology Department of Biology Friedrich‐Alexander‐Universität (FAU) Erlangen‐Nürnberg 91058 Erlangen Germany; ^2^ Erlangen National High Performance Computing Center (NHR@FAU) 91058 Erlangen Germany; ^3^ Institute of Applied Optics and Biophysics Friedrich‐Schiller University Jena 07743 Jena Germany; ^4^ Department Biophysical Imaging Leibniz Institute of Photonic Technologies e.V. 07745 Jena Germany; ^5^ Division of Biophysics Research Center Borstel Leibniz Lung Center 23845 Borstel Germany; ^6^ Centre for Structural Systems Biology (CSSB) 22607 Hamburg Germany; ^7^ Jena Center for Soft Matter 07743 Jena Germany; ^8^ FAU Profile Center Immunomedicine (FAU I‐MED) 91054 Erlangen Germany

**Keywords:** antimicrobial resistance, antimicrobial peptides, bacterial membrane, molecular dynamics simulation, machine learning, plasma membrane, peptide design

## Abstract

The United Nations have committed to end the epidemics of communicable diseases by 2030 (SDG Target 3.3). In contrast with this ambition, the rise of Multi Drug Resistant (MDR) and Pan Drug Resistant (PDR) bacteria poses a threat of a return to the pre‐antibiotic era. It is of high priority to find new therapies that target the ESKAPEE group of pathogens and their drug‐resistant strains. Antimicrobial peptides (AMPs) are an emerging class of antibiotics that hold promises of overcoming bacterial resistance by using both novel mechanisms of action as well as targeting already known pathways. The chemical space of AMPs is potentially huge and methodologies allowing the rational exploration of novel structures are highly needed. This review focuses on case studies that give novel insights about the mechanisms of action, resistance and selectivity of some relevant AMPs, exemplifying the importance of microscopic, computational and experimental tools. Particular focus will be devoted to bacterial membranes and how AMPs can target them while sparing human plasma membranes, in order to become safer drugs. The lessons learned from the literature cases give directions toward the development of AMPs as drug products.

## Graphical abstract

Antimicrobial peptides (AMPs) are promising candidates for next‐generation antibiotics, acting through mechanisms such as membrane disruption and intracellular targeting. This review examines how variations in bacterial membrane composition critically influence AMP activity. Through selected case studies, it elucidates key structure‐activity relationships and modes of action. The insights gained support the rational design of AMPs as innovative therapeutics, addressing the pressing global demand for new antimicrobial strategies amid rising antibiotic resistance.

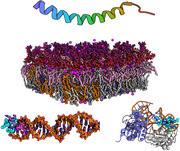

## 1 Introduction

Nearly 80 years after the widespread introduction of antibiotics, the rapid rise of drug‐resistant infections urgently calls for the development of novel antimicrobial therapies. Addressing bacterial infections through innovative preventive and curative approaches is among the United Nations targets for sustainability. It is paramount to identify treatments that minimize the risk of evolving resistance, ensuring long‐term efficacy.

Among the emerging therapeutic strategies, antimicrobial peptides (AMPs) have shown great potential to overcome and mitigate drug resistance mechanisms.^[^ ^1^ ^]^ AMPs represent a diverse class of molecules unified by their primary biological activity: the ability to kill bacteria and other microorganisms, including fungi, viruses, and parasites.^[^ ^1^ ^]^ These peptides exhibit remarkable structural diversity, characterized by variations in amino acid composition and secondary structure, often displaying some degree of structural plasticity.^[^ ^2^ ^]^ For instance, data from the Antimicrobial Peptide Database (APD,^[^ ^3^ ^]^ link to the statistics: https://aps.unmc.edu/facts) reveal that most AMPs are relatively short, with a length distribution centered around 30 amino acid residues, with a long tail extending toward larger proteins like lysozyme (14 kDa) and lactoferrin (80 kDa). AMPs are typically cationic and hydrophobic, with a net positive charge around +3*e*_*o*_ and a hydrophobic residue content of approximately 50%. The diverse chemical space of AMPs reflects the variety of organisms that produce them — ranging from mammals and insects to plants, amphibians, and microorganisms — as well as the wide heterogeneity of bacterial species and strains they target. AMPs are part of the innate immune response in many organisms or may serve as a competitive mechanism in microbial ecosystems.^[^ ^4^ ^]^

This review aims to identify and predict trends in AMP discovery by examining both historical and contemporary approaches. The discovery of AMPs dates back to 1922 when Sir Alexander Fleming serendipitously identified lysozyme and its antimicrobial activity. Early AMP discoveries, such as Colistin, Daptomycin, and Gramicidin, came from painstaking isolation efforts from soil samples.^[^ ^5^, ^6^, ^7^ ^]^ However, the clinical development of AMPs has been marked by alternating periods of success and challenge, often due to the balance between efficacy and side effects,^[^ ^5^, ^8^ ^]^ caused by the peptides' suboptimal pharmacokinetic (PK) properties.^[^ ^9^, ^10^, ^11^, ^12^ ^]^ The technological limitations of early peptide synthesis, combined with a lack of understanding of key concepts such as structure‐toxicity relationships (STR), structure‐activity relationships (SAR), and PK, further hindered their development. As a result, from the 1960s to 2000, peptide therapeutics, including AMPs, as a whole were still underrepresented in the clinic. AMPs were largely overshadowed by small‐molecule antibiotics, which were cheaper and more easily administered orally.

However, since the early 2000s, there has been a sharp increase in the number of peptides entering clinical trials and gaining approval,^[^ ^13^, ^14^ ^]^ supported by advances in peptide synthesis and a better understanding of their pharmacological properties. This review will illustrate how, today, AMP research benefits from advances in both experimental and computational tools, enabling a transition from serendipitous discoveries to structure‐guided rational design. In addition, the discovery of novel AMPs can take advantage of the co‐evolution between AMPs and bacteria, and use these structural scaffolds to obtain novel peptide drugs.^[^ ^15^ ^]^ The integrated use of experimental and computational approaches provides a powerful framework for exploring AMP structure and function, offering the potential to drive the discovery of new, effective antimicrobial treatments.

## 2 Mechanisms of Action of Antimicrobial Peptides

**Figure 1:**
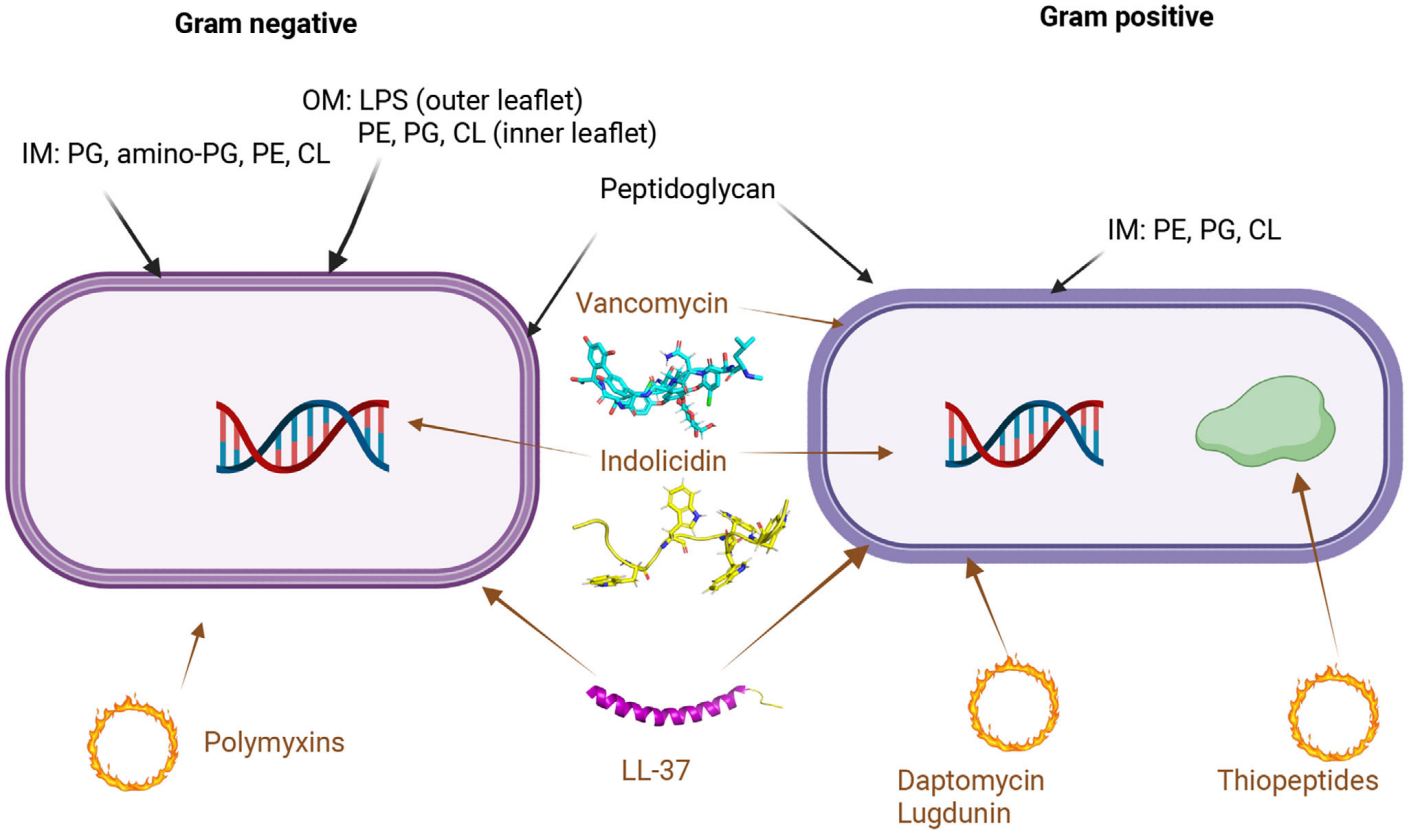
Distinct AMPs and their mechanisms of action. Vancomycin targets cell wall synthesis specifically in Gram‐positive bacteria; Indolicidin exhibits broad‐spectrum activity by inhibiting DNA synthesis; LL‐37 disrupts bacterial membranes in both Gram‐positive and Gram‐negative bacteria; Polymyxins are LPS‐ and membrane‐active, effective against Gram‐negative bacteria; Daptomycin and lugdunin target the membrane of Gram‐positive bacteria; Thiopeptides inhibit bacterial protein synthesis. Abbreviations: OM – Outer Membrane, IM – Inner Membrane, LPS – Lipopolysaccharide, PG – Phosphatidylglycerol, PE – Phosphatidylethanolamine, CL – Cardiolipin.

As outlined in the Introduction, AMPs represent a diverse class of molecules with distinct structural features and mechanisms of action. **Figure** **1** illustrates how different AMPs target specific bacterial cell structures, classifying them according to their primary cellular targets: membranes, cell walls, or intracellular components such as DNA, RNA, and proteins. Beyond these three major categories, this review also includes peptides that specifically target bacterial biofilms, which form complex supra‐cellular structures.

This section examines how computational and experimental approaches complement one another to unravel the intricate mechanisms underlying the activity and selectivity of AMPs, providing a foundation for the rational design of novel antimicrobial agents.

We begin with an overview of the computational and experimental techniques most commonly employed over the years to investigate AMP mechanisms of action, as highlighted in the case studies presented. Although there are some specialized methods for specific applications, this review focuses on the most widely utilized and impactful techniques.

**Molecular dynamics simulations**: Molecular dynamics (MD) simulations act as a computational microscope, enabling detailed investigations of molecular systems at spatial and temporal scales often beyond the reach of experimental techniques.^[^ ^16^ ^]^ Recent advances in computational power, algorithm development, and the growing availability of high‐resolution data, such as lipidomics and protein structure models, have facilitated the simulation of increasingly complex and realistic systems.^[^ ^16^, ^17^ ^]^

A key challenge in MD simulations lies in the accurate parameterization of force fields, which define the (non‐)bonded interactions between atoms. Classical MD simulations are limited in their ability to model chemical reactions, such as bond breaking or formation. However, recent innovations, including constant‐pH MD techniques, now enable the simulation of dynamic (de)protonation events of proteins and lipids under varying pH conditions.^[^ ^18^ ^]^ MD simulations are computationally demanding, often requiring access to high‐performance computing (HPC) facilities. To mitigate these demands, researchers frequently employ coarse‐grained force fields, such as MARTINI^[^ ^19^, ^20^, ^21^, ^22^ ^]^ and SIRAH,^[^ ^23^ ^]^ which simplify systems by grouping atoms into larger “super‐beads”. This approach reduces the degrees of freedom and computational complexity, while still capturing essential system dynamics. Atomistic force fields like CHARMM^[^ ^24^, ^25^, ^26^, ^27^ ^]^ and AMBER^[^ ^28^, ^29^, ^30^ ^]^ remain indispensable for highly detailed simulations, albeit with higher computational costs.

**Machine learning**: Machine learning (ML) has become a powerful bioinformatics tool for classifying, predicting, and designing novel AMPs. By identifying patterns in training data, ML methods generate new peptide sequences with potential antimicrobial activity. Common techniques include support vector machines (SVMs), neural networks (NNs), discriminant analysis (DA), random forests (RF), hidden Markov models (HMMs), and long short‐term memory (LSTM) models.

These methods vary in their dataset requirements, interpretability, and ability to generate novel sequences. Simpler models, such as RF, DA, SVM, and HMMs, perform well with small to medium‐sized datasets, while more complex approaches like NNs and LSTMs require larger datasets and longer training times to avoid overfitting and limited sequence novelty.

A critical challenge across all ML approaches is the scarcity of standardized, high‐quality experimental data from diverse sources. AMP datasets are often small, which constrains the capabilities of advanced models. Furthermore, the lack of pharmacokinetic (PK) and toxicological data limits the clinical translation of *in vitro* active peptides.

Despite these limitations, ML methods excel in rapidly identifying active candidates, as demonstrated in recent studies.^[^ ^31^ ^]^ However, designing AMPs that overcome inherent challenges, such as poor PK profiles, remains a significant hurdle, highlighting the need for more robust datasets and advanced modeling strategies.

**Fluorescence microscopy**: Imaging of specific bacterial structures through fluorescence labeling. The huge advantage is the applicability to both fixed and live material. Spatial resolution of conventional approaches such as wide‐field, confocal, two‐photon or TIRF and lightsheet microscopy is down to 200 nm, i.e. on the scale of sub‐cellular bacterial structures, and down to the molecular level (individual proteins and molecular aggregates) through super‐resolution microscopy approaches such as STED, STORM/PALM, SIM or MINFLUX microscopy, and dynamics can be recorded with milli‐second to second time resolution, depending on the method in hand.^[^ ^32^ ^]^ Advantages are the molecular specificity and possibility to observe both fixed and live bacteria. Disadvantages are the need for fluorescence staining, increasing sample preparation complexity, and the potential phototoxicity on the sample as well as photobleaching of the labels, limiting recording times.

**Electron microscopy (EM) and X‐Ray or XUV microscopy**: Imaging of bacterial structures using electron beams (EM) or X‐ray and XUV radiation. The big advantage of these microscopy techniques is the high spatial resolution, being able to resolve sub‐cellular features down to the molecular level and even highlight structural details of (macro)molecules.^[^ ^33^, ^34^, ^35^ ^]^ The disadvantage are the need for complex instrumentation performing in vacuum, accurate sample fixation and preparation (such as cryo‐fixation), making live‐cell measurements impossible, and intense data analysis.

**Raman microscopy**: Label‐free molecular fingerprints of a bacterial sample. Raman spectra allow to identify certain molecular patterns of a sample like inflammation or infection states of samples or differences in molecular compositions or status of bacteria.^[^ ^36^ ^]^ The big advantage of Raman microscopy is its label‐free use, i.e. it can be employed directly to a sample of interest such as a patient sample or bacterial colony. The disadvantage is a lower spatial and temporal resolution than other microscopy techniques (which is still on the sub‐bacterial scale) and a rather low signal yield with low signal‐to‐noise levels, which is tackled with non‐linear and multimodal Raman approaches.^[^ ^37^ ^]^

**Atomic force microscopy (AFM)**: Scanning of a sample surface allows the characterization of its properties down to the molecular level, such as surface height profile, stiffness or molecular interaction forces.^[^ ^38^, ^39^ ^]^ The advantage of AFM is its high spatial resolution and high information content, while disadvantages involve limitation to surfaces only and the use of tips that could potentially influence the sample.

**Nuclear magnetic resonance (NMR)**: A key tool for resolving structures of a molecules such as AMPs.^[^ ^40^ ^]^ Its advantage relies on a strong chemical and structural characterization of molecular structures, yet it requires large instrumentation with isolated and well prepared molecular samples and intense data interpretation.

**Enzyme‐linked Immunosorbent Assay (ELISA), quartz crystal microbalance with dissipation monitoring (QCM‐D) and related techniques**: These techniques allow to explore binding affinities between molecules such as AMPs on surfaces or membranes with greatest details.^[^ ^41^ ^]^

### 2.1 Membrane‐Active AMPs

The majority of the AMPs currently undergoing clinical trials exert their antimicrobial effects by damaging the bacterial membrane.^[^ ^42^ ^]^ Since membrane‐targeting AMPs rather address general physico‐chemical properties of membranes (charge, hydrophobicity) than specific structural motifs, they offer a promising strategy to bypass the resistance problem that plagues many conventional antibiotics. The compositional differences and thus the different properties of Gram‐negative and Gram‐positive bacterial membranes are reflected in the structural diversity of membrane‐active AMPs.

**Figure 2:**
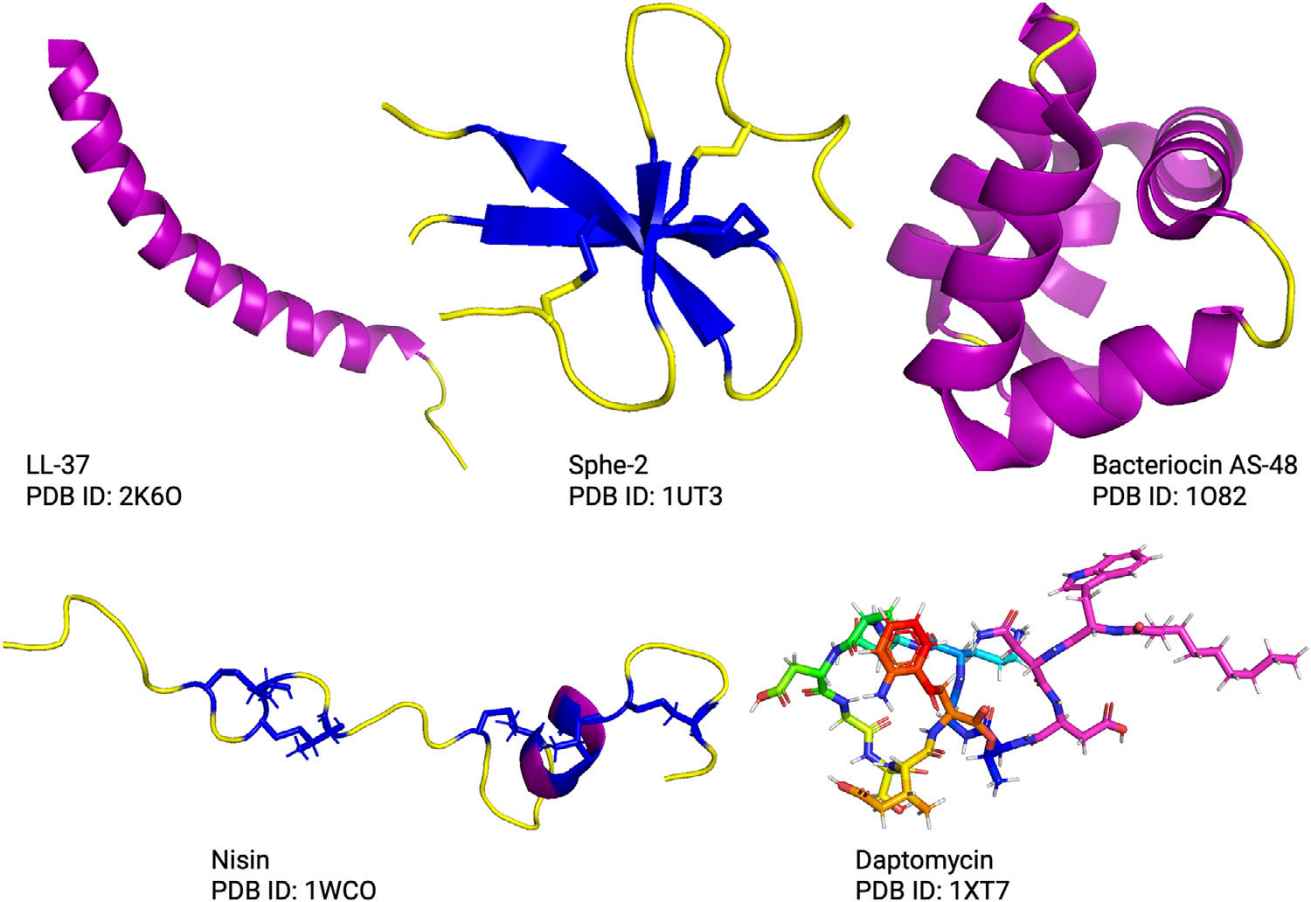
Examples of membrane‐active peptides with distinct structural motifs. LL‐37 features a mono‐α‐helical structure; Sphe‐2 (king penguin β‐defensin) has a defensin‐like structure stabilized by multiple disulfide bridges; Bacteriocin AS‐48 exhibits a globular protein structure characterized by several α‐helices; Nisin includes multiple macrocycles tethered by lanthionine monosulfide bridges (highlighted in *blue*); Daptomycin has a non‐macrocyclic region (shown in *magenta*) connected to a macrocyclic scaffold.

**Figure 3:**
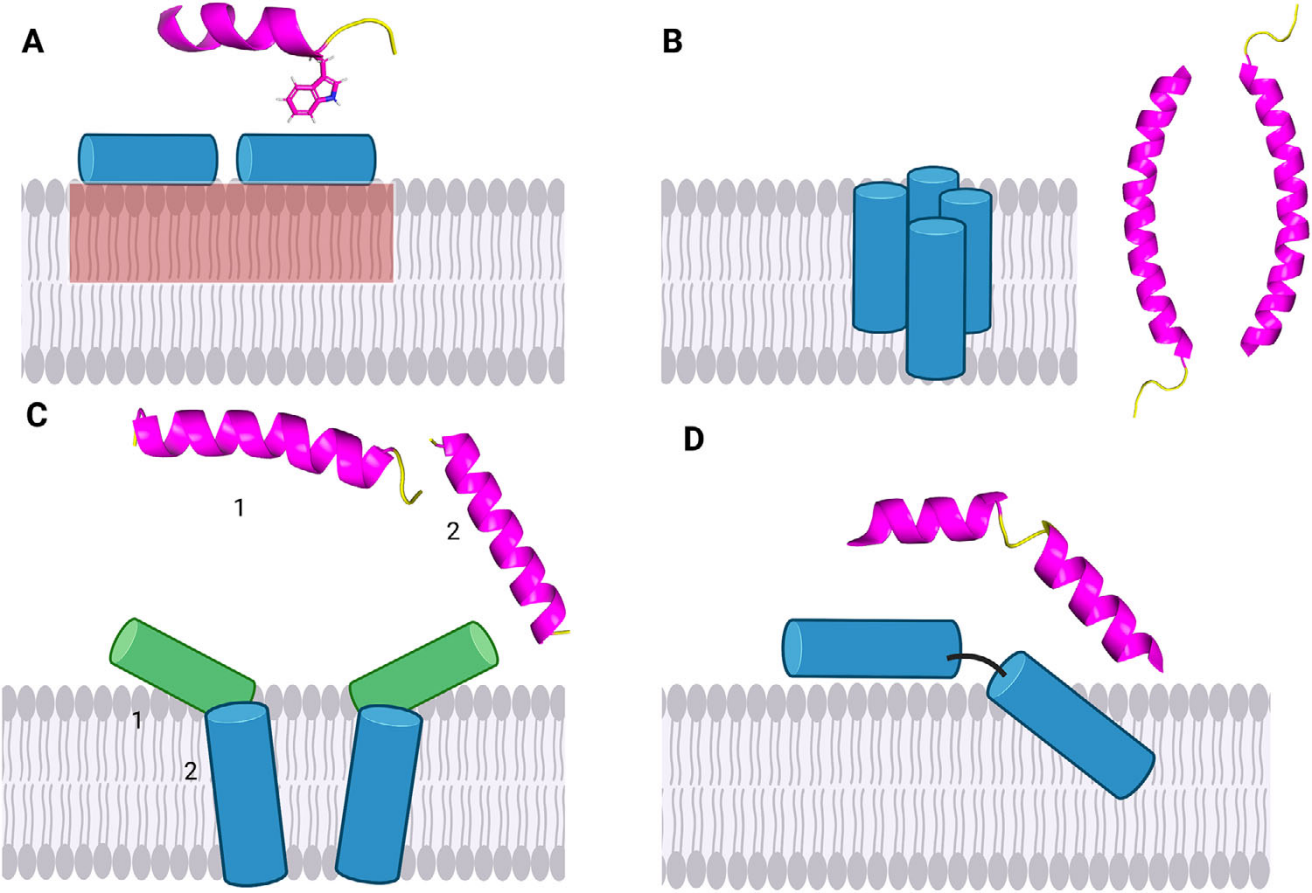
Mechanisms of membrane disruption by various AMPs. A) Carpet‐like mechanism, exemplified by indolicidin (P→A variant, PDB ID: 1hr1). The red rectangle indicates the formation of peptide‐induced lipid domains, which may contribute to membrane destabilization. B) Homo‐oligomerization (with parallel or antiparallel peptide alignment) and toroidal pore formation, as observed for LL‐37 (PDB ID: 2k6o). C) Heterodimer formation and toroidal pore formation, illustrated by Magainin‐2 (1, PDB ID: 2mag) and PGLa (2, predicted by PepFold4,^[^ ^85^ ^]^ pH 7). D) Helix‐kink‐helix motif binding and membrane insertion, demonstrated by Melittin (PDB ID: 2mlt). In the peptide molecular structures shown for each mechanism, α‐helices are rendered in *magenta* and random coils in *yellow*. In the schematic membrane interaction models, green and blue cylinders denote the helical segments of the peptides.

**Figure** **2** shows five well‐studied membrane‐active AMPs, all of which form membrane pores but through distinct mechanisms.^[^ ^43^, ^44^, ^45^, ^46^, ^47^, ^48^ ^]^ These include peptides with entirely different 3D structures, emphasizing the versatility of AMPs in the targeting of bacterial membranes. **Figure** **3** illustrates four representative membrane‐disruptive mechanisms employed by AMPs, highlighting their structural diversity and implications for selectivity and resistance. Panel 3A depicts a carpet‐like model, exemplified by Trp‐ and Arg‐rich amphipathic peptides such as indolicidin, which localize at the membrane interface through hydrophobic and electrostatic interactions.^[^ ^49^ ^]^ Peptide binding may induce lipid domain formation (red rectangle), destabilizing membrane integrity and promoting permeabilization.^[^ ^49^, ^50^ ^]^ Panel 3B shows how helical peptides like LL‐37 form homo‐oligomeric toroidal pores that span the membrane, with pore size and peptide stoichiometry influenced by membrane thickness.^[^ ^51^ ^]^ Panel 3C highlights the cooperative action of Magainin‐2 and PGLa, which form heterodimeric pores stabilized by N‐ to C‐terminal salt bridges.^[^ ^52^, ^53^ ^]^ Panel 3D features Melittin's helix‐kink‐helix motif, where the hydrophobic N‐terminus inserts into the membrane while the cationic C‐terminus remains surface‐bound. The kink, introduced by Proline and Threonine residues, may facilitate a transition toward pore structures similar to those in panel B.^[^ ^54^, ^55^, ^56^, ^57^ ^]^

AMPs that target bacterial membranes vary widely in their composition, and may feature non‐canonical amino acids or macrocyclic scaffolds, further expanding the AMP chemical space. For example, Nisin and Daptomycin, as shown in Figure 2, both contain non‐canonical residues and possess macrocyclic structures. These macrocyclic architectures can endow AMPs with unique mechanisms of action, such as the ability to chelate cations (as seen in Daptomycin) or form nanotubes within bacterial membranes (as demonstrated for Gramicidin A and Lugdunin). Understanding these varied mechanisms through experimental and computational studies is essential for the design of new AMPs with improved efficacy and reduced potential for resistance development. In the following Sections, we will focus on AMPs that target bacterial membranes.

#### 2.1.1 LL‐37: Tetrameric Membrane Pores Highlight the Importance of Peptide Cooperativity

LL‐37, the active form of the cathelicidin antimicrobial peptide (CAMP), is derived from the proteolytic cleavage of the inactive precursor CAP‐18 (18 kDa). LL‐37 adopts an amphipathic α‐helical structure,^[^ ^58^ ^]^ and at neutral pH, it spontaneously forms oligomers in solution.^[^ ^59^ ^]^ To understand how LL‐37 interacts with bacterial membranes, Zhao et al.^[^ ^44^ ^]^ employed the GROMOS united‐atom force field to model the interactions of LL‐37 monomers with simplified, homogeneous membranes composed of POPG (representing bacterial membranes) and POPC (representing mammalian membranes). Their findings demonstrated that LL‐37 interacts with both types of membranes, but the interaction with POPC was much weaker, and resulted in partial helix unfolding.^[^ ^44^ ^]^

A combined experimental and computational study additionally took into account the quaternary structure of LL‐37 to investigate LL‐37's channel‐forming properties.^[^ ^58^ ^]^ Using X‐ray crystallography, a tetrameric conformation of LL‐37 was observed (PDB ID 7pdc),^[^ ^58^ ^]^ which was consistent with circular dichroism experiments indicating oligomerization and toroidal pore formation.^[^ ^59^ ^]^ The experimentally determined tetramer structure was used in molecular dynamics (MD) simulations embedded within a model of the Gram‐negative inner membrane (POPE:POPG at a ration of 3:1) and homogeneous bilayers of POPE, POPG, and POPC. The tetramer was more stable in the Gram‐negative inner membrane than in POPC, which explains LL‐37's selectivity for bacterial membranes over mammalian ones.^[^ ^58^ ^]^

LL‐37 is one of the best‐studied α‐helical AMPs, and its potent antimicrobial activity and safety has been evaluated in clinical trials.^[^ ^60^, ^61^ ^]^ Its therapeutic potential has driven the synthesis of various LL‐37 derivatives. Studies on shorter fragments, such as the first 20 or 32 residues, revealed that residue R23 plays a crucial role in membrane anchoring, while the C‐terminal region is essential for dimerization and deep insertion into the membrane.^[^ ^62^, ^63^ ^]^ Truncation of the C‐terminus causes a drastically changed action mode,^[^ ^62^ ^]^ with LL‐37 forming toroidal pores, whereas LL‐32 destabilizes membranes through a carpet‐like mechanism. Figure 3 illustrates that perfectly helical peptides, such as LL‐32, do not form toroidal pores, highlighting the complexity of peptide structure‐function relationships. Minor structural modifications can result in substantial changes in how a peptide interacts with bacterial membranes. Moreover, LL‐37 and its derivatives have been demonstrated to posses a multi‐factorial antimicrobial activity, including endotoxin neutralization and immune modulation roles. The endotoxin‐neutralizing effect was demonstrated for the truncated version LL‐32, leading to reduced TLR‐4 signaling by inhibiting the TLR‐4 activation pathway shown in Figure 10, and resulting in decreased TNF‐α secretion.^[^ ^64^ ^]^ The anti‐inflammatory activity of LL‐32 was also attributed to its reorganizing activity on host cell membrane raft domains, reducing overall TLR‐4 activation.^[^ ^64^ ^]^ Overall the studies regarding LL‐37 and its close derivatives highlight the complexity of its mechanism of action and the challenges in reconstructing the SAR of a multi‐factorial AMP.

**Figure 4:**
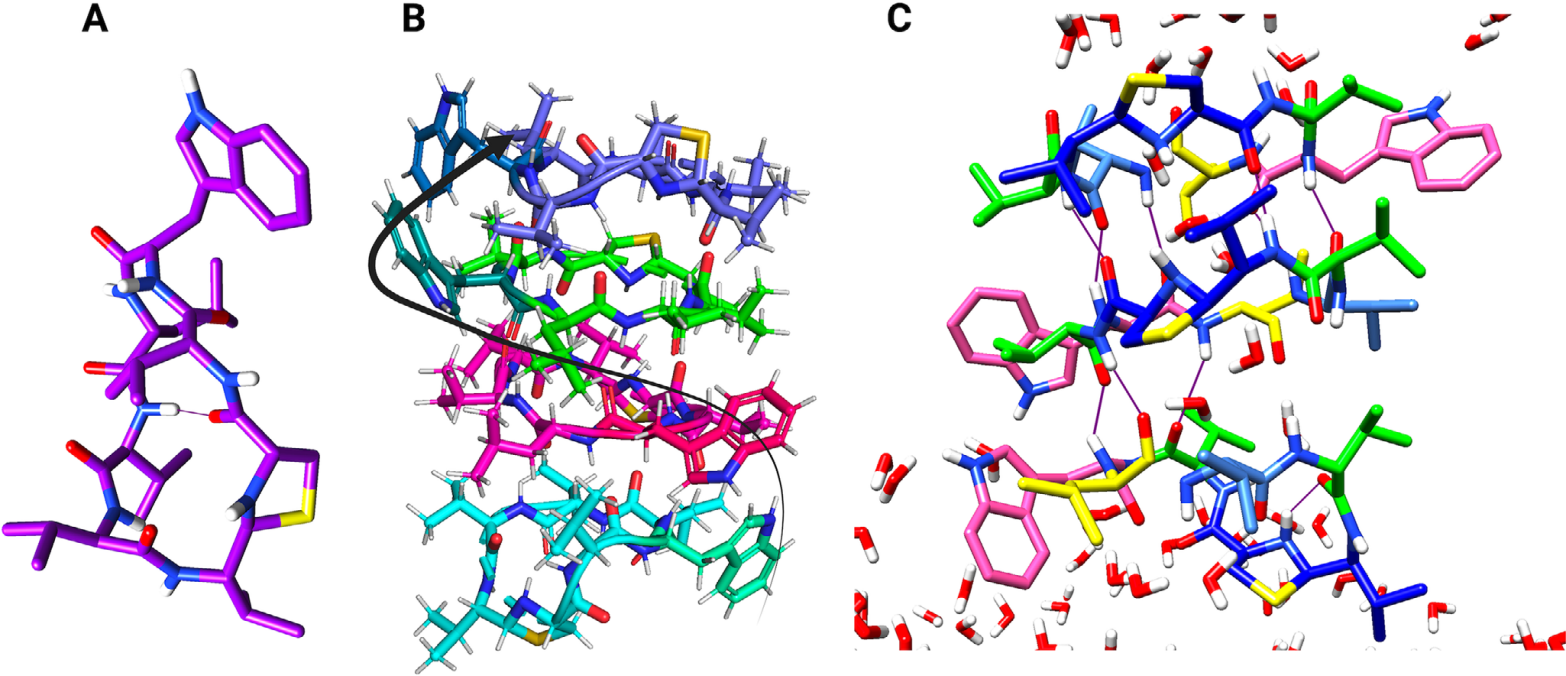
Structural insights into lugdunin and its membrane interaction. A) Minimized 3D structure of lugdunin; B) Lugdunin stacks in the trans‐conformation, with tryptophan residues oriented to minimize steric hindrance; C) Lugdunin pore formation within a DMPC membrane: the number of stacked lugdunin molecules varies based on membrane thickness (Colors: Leucine in *yellow*, valine in *green*, tryptophan in *pink*, thiazolidine in *blue*).

**Figure 5:**
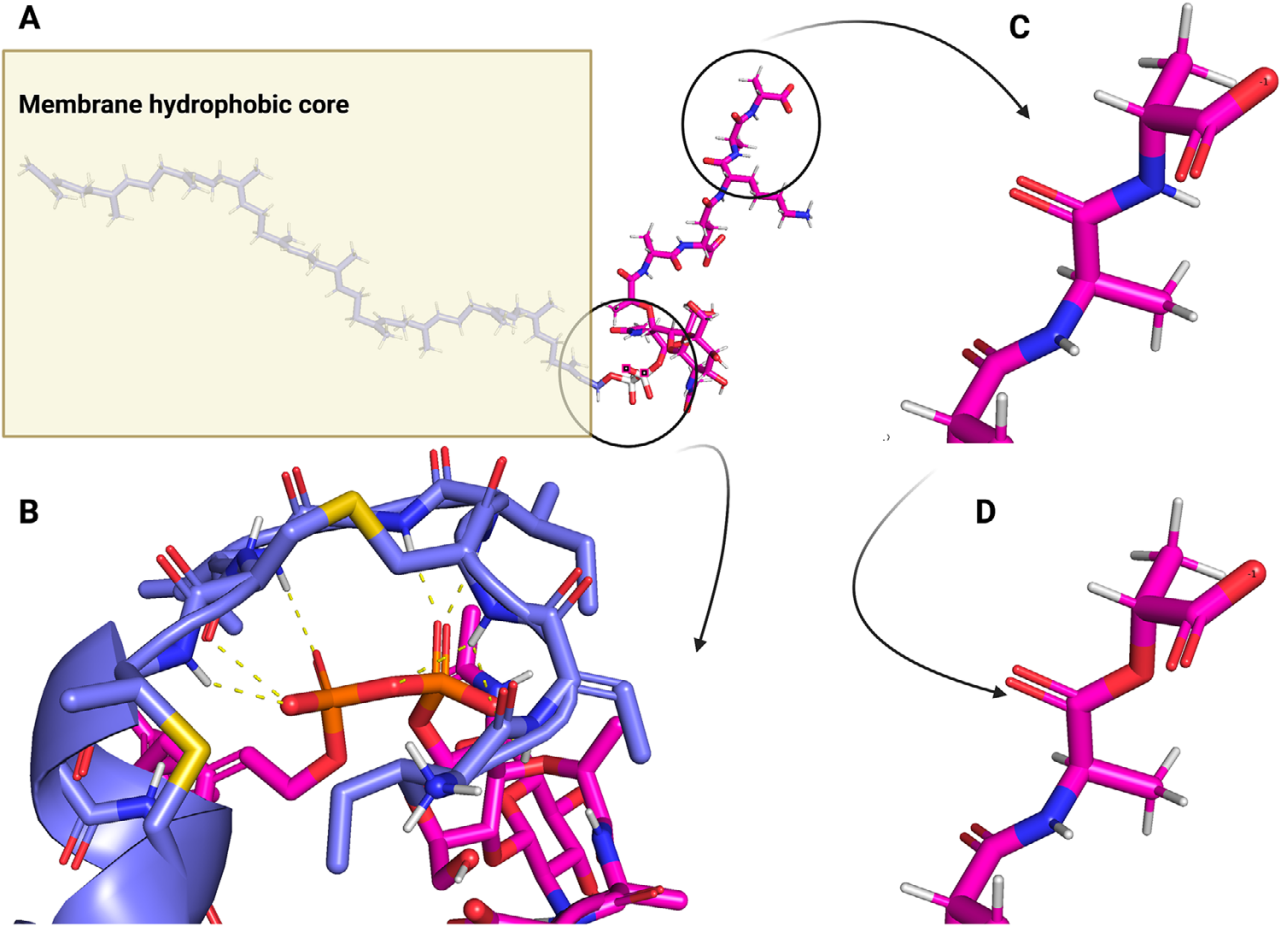
Interaction sites of lipid II. A) Structure of lipid II; (B) Nisin binding to the pyrophosphate group of a lipid II mimetic (zoomed view from PDB ID: 1wco^[^ ^134^ ^]^); (C) Close‐up of the D‐Ala‐D‐Ala dipeptide in lipid II, the binding site recognized by vancomycin and other glycolipids; (D) Mutated dipeptide form D‐Ala‐D‐Lactate, where replacing the amide nitrogen with an oxygen atom disrupts one hydrogen bond with vancomycin, causing a sharp decrease in binding affinity.

**Figure 6:**
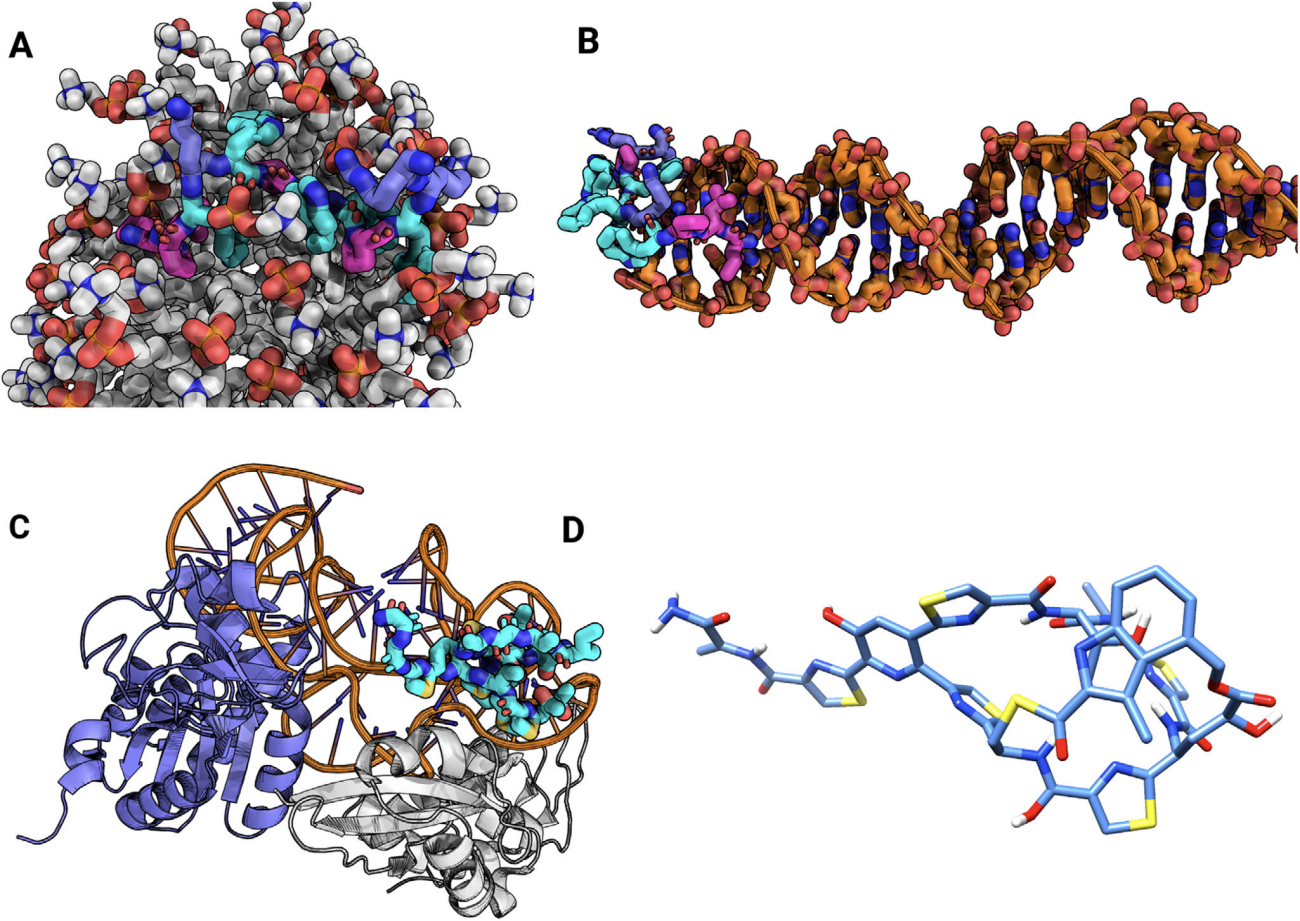
Structure and interactions of indolicidin and thiopeptides with cellular targets. A) Indolicidin peptide (PDB ID: 1g89) interacting with a DPC (dodecylphosphocholine) micelle (color coding: basic residues (Arg, Lys): blue, Tryptophan: cyan, hydrophobic residues: magenta, DPC: grey. Phosphate atoms in orange, Oxygen in red and Nitrogen in blue);^[^ ^160^ ^]^ B) Interaction model of indolicidin with DNA (color coding: basic residues (Arg, Lys): blue, Tryptophan: cyan, hydrophobic residues: magenta, ds‐DNA: orange. Phosphate atoms in orange, Oxygen in red and Nitrogen in blue); C) Thiostrepton (*cyan*) complexed with the 50S ribosomal protein L10 (*blue*), 50S ribosomal protein L11 (*grey*), and 23S ribosomal RNA (*orange* backbone) (PDB ID: 5d8h); D) Nosiheptide (PDB ID: 2zjp), a 26‐membered thiopeptide related to micrococcin and thiostrepton, showing the rigid macrocyclic scaffold and abundance of aromatic rings.

**Figure 7:**
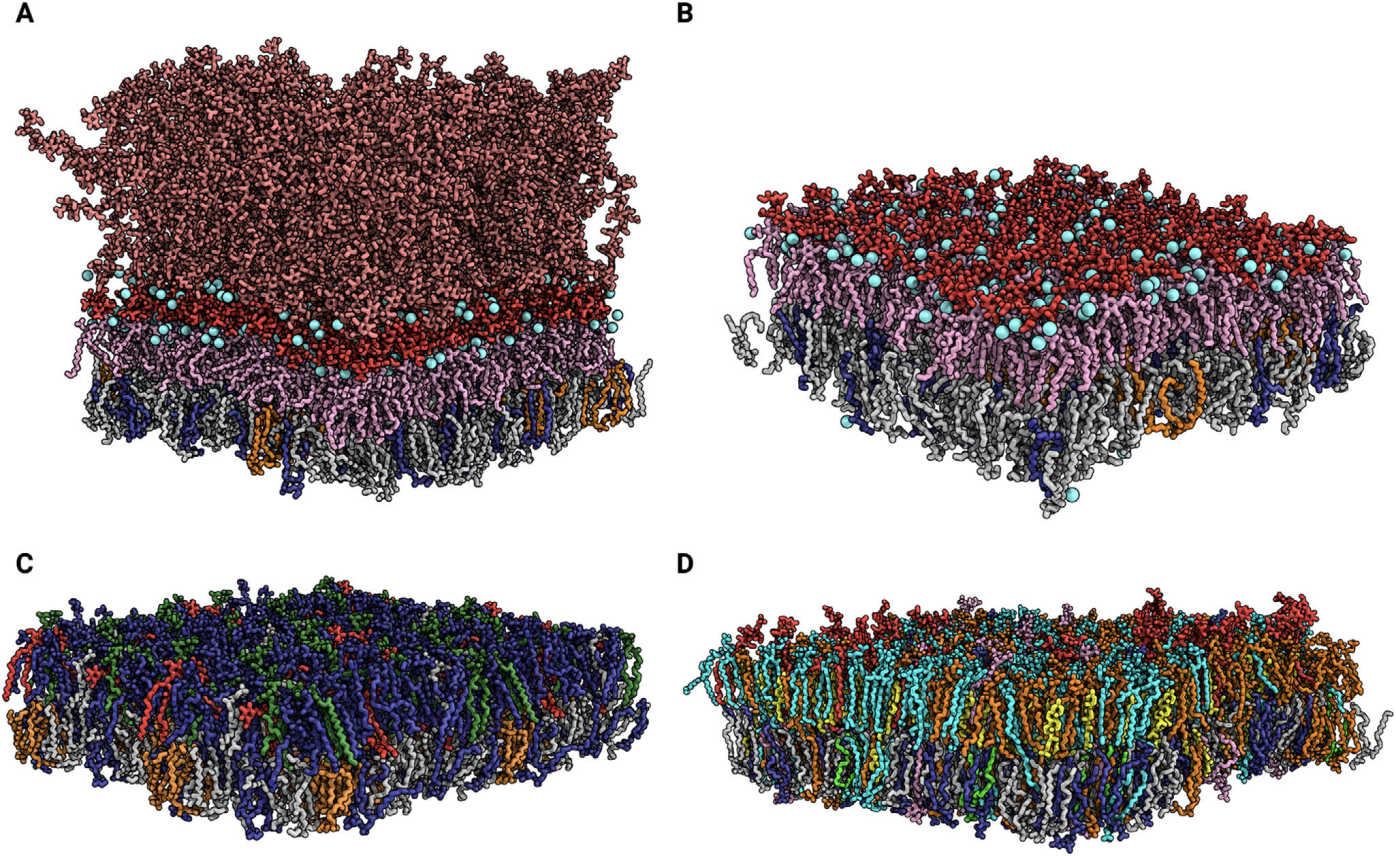
Membrane structures of different cell types. A) *E. coli* outer membrane with O‐antigen; B) *E. coli* deep rough mutant (Re) outer membrane; C) Gram‐positive membrane with *B. subtilis*‐like asymmetric composition; D) Red blood cell (RBC) membrane. Color coding: *Red* for glycolipids; LPS: *Pink* for (Lipid A), *Bright red* for negatively charged sugars, *Salmon* for neutral sugars; *Grey* for PE; *Deep blue* for anionic lipids — PG in bacterial membranes and PS in RBCs; *Orange* for cardiolipin; *Bright orange* for PC; *Forest green* for lysyl‐PG; *Sand* for cholesterol; *Bright green* for PA; *Pink* for PI; *Cyan* for SM. Calcium ions are represented as *cyan* spheres. Sodium, chloride, and water are omitted for clarity.

**Figure 8:**
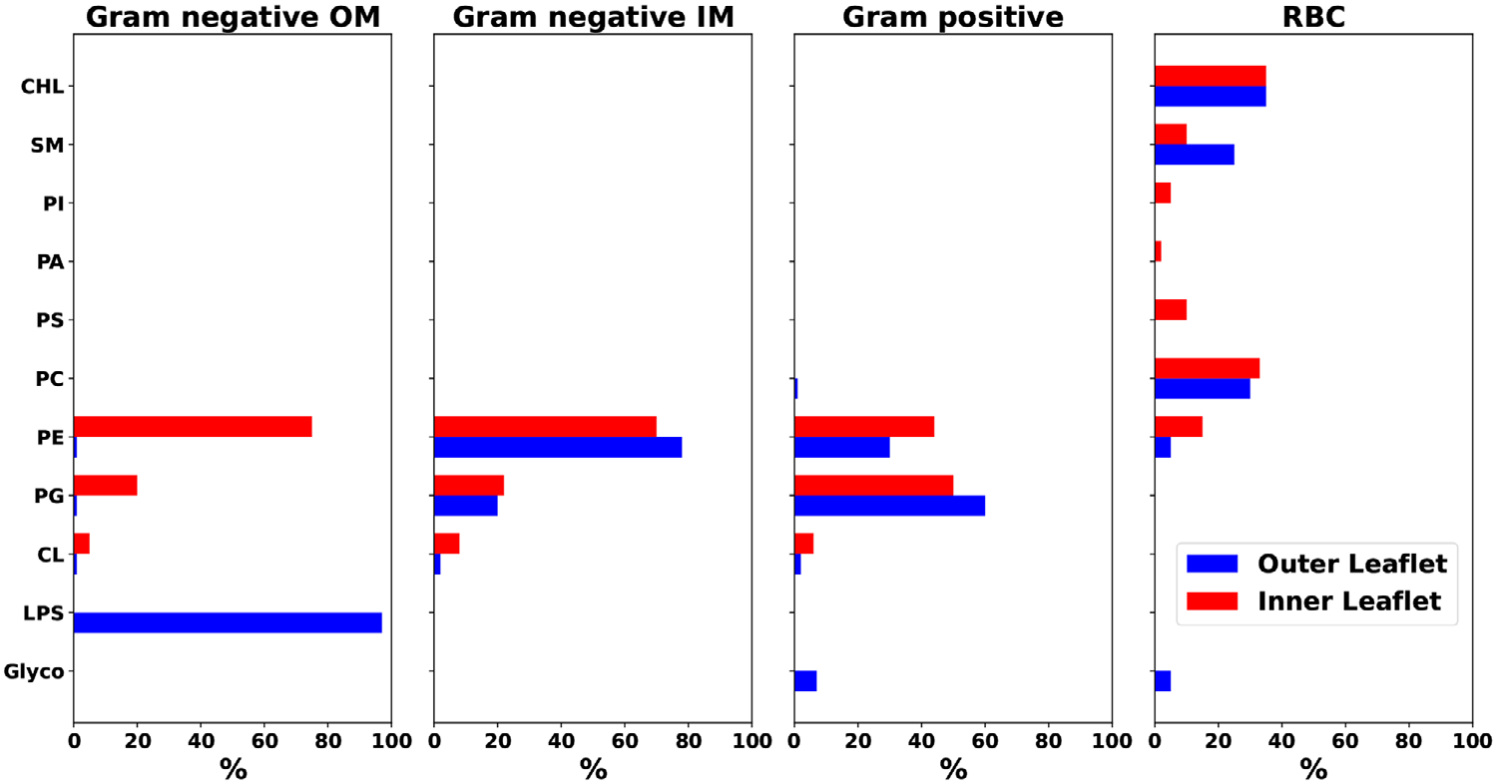
Approximate membrane compositions of different cell types. Gram‐negative outer membrane, Gram‐negative inner membrane, Gram‐positive cell membrane, and red blood cell membrane. *Blue* bars indicate the outer leaflet composition, while *red* bars represent the inner leaflet composition.

**Figure 9:**
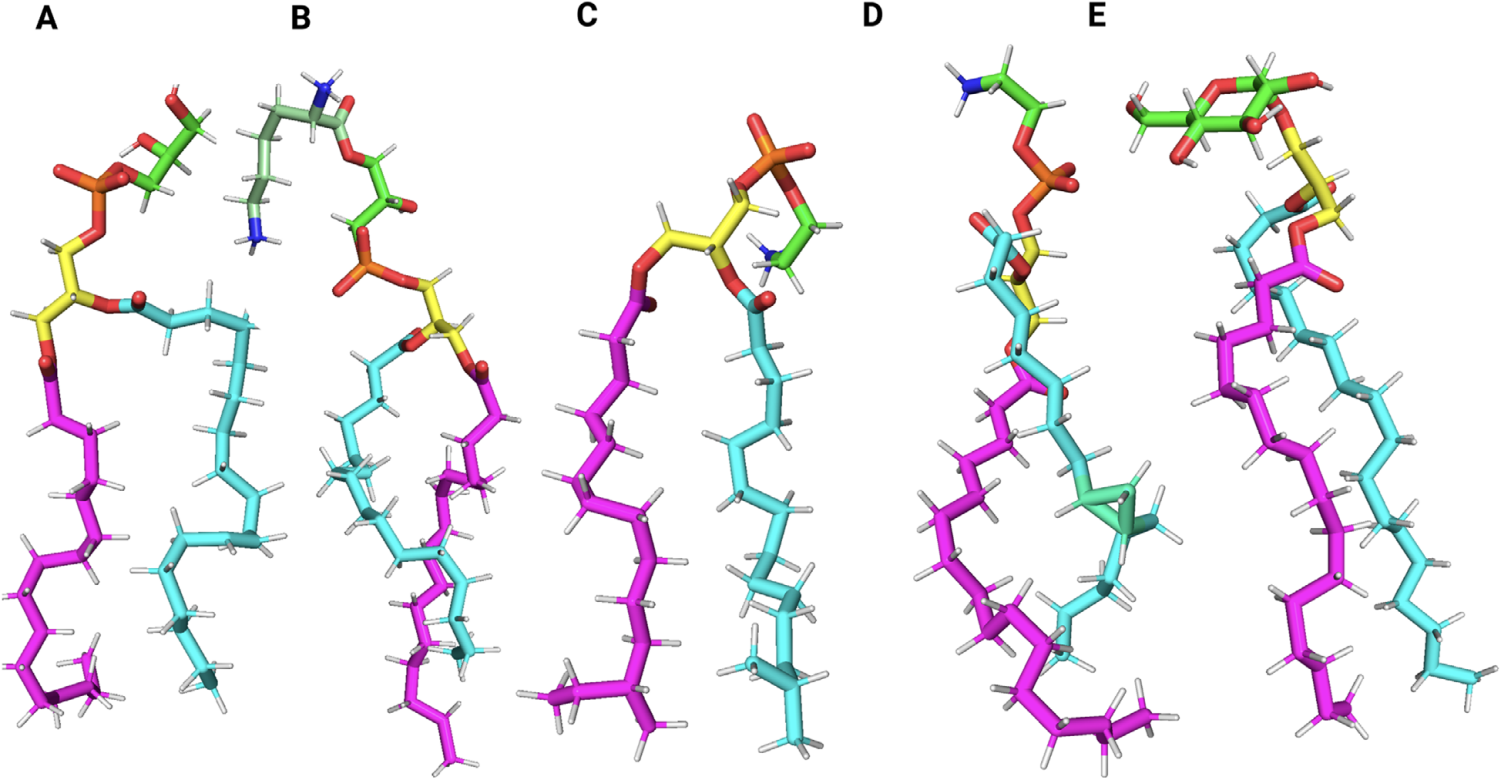
Key phospholipids and glycolipids in bacterial membranes. A) Phosphatidylglycerol (PG); B) Lysyl‐PG; C) Phosphatidylethanolamine (PE) with iso‐ (*cyan*) and anteiso‐ (*magenta*) acyl chains; D) PE with a cyclopropanated acyl chain (*cyan*, with cyclopropane highlighted in *turquoise*); E) Diacyl‐glucosyl‐glycerol, the most common and simplest glycolipid found in Gram‐positive bacteria. Carbon atom color scheme: Headgroups in *green*, glycerol backbone in *yellow*, *sn‐1* acyl chain in *magenta*, and *sn‐2* acyl chain in *cyan*.

**Figure 10:**
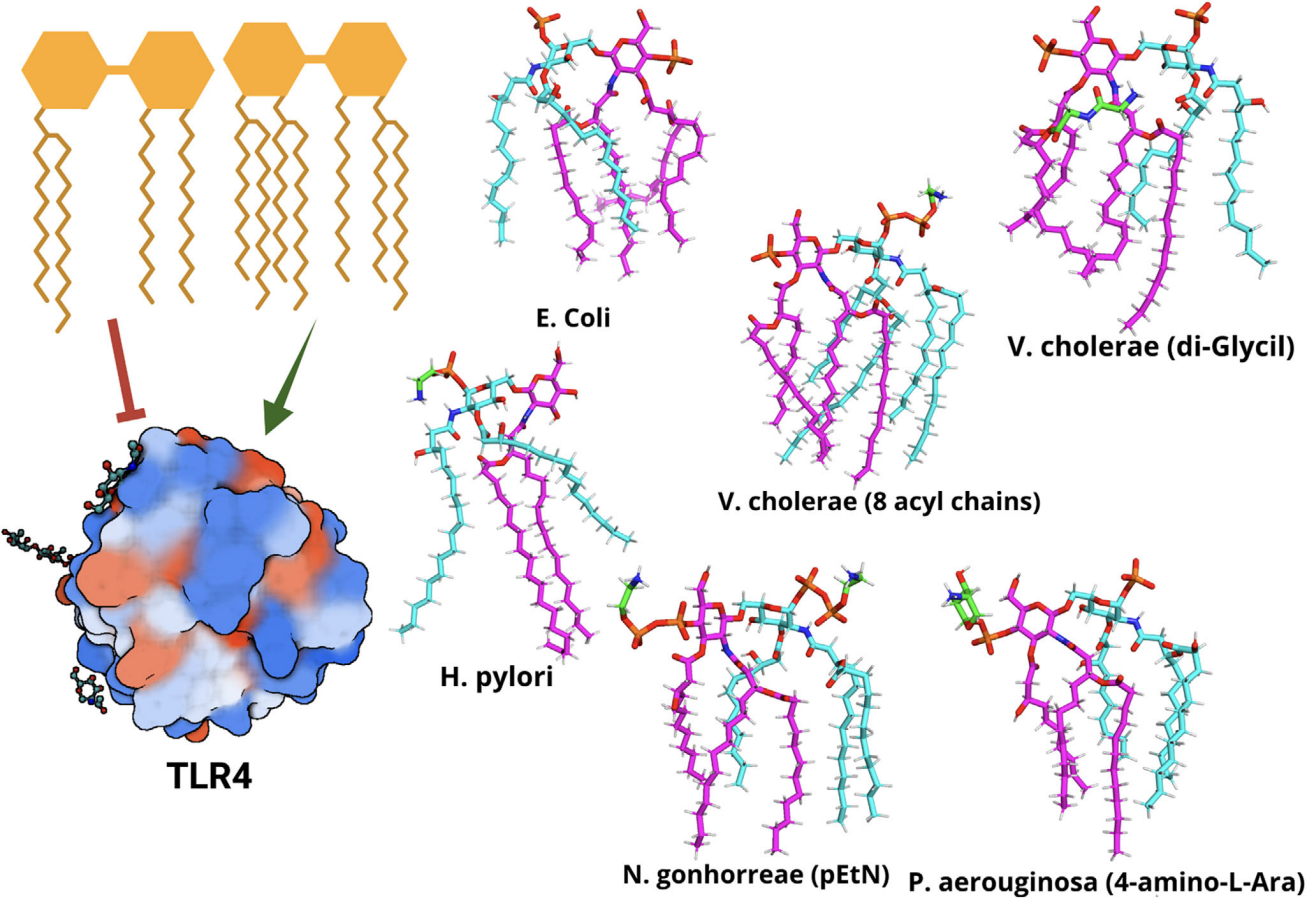
Structural variability of Lipid A and its role in modulating the innate immune response. The structure of Lipid A, which can vary significantly across bacterial species, is crucial in interacting with the Toll‐like receptor 4 (TLR4 structure, PDB ID: 2z65). Lipid A may be tetra‐acylated (e.g., *H. pylori*), hexa‐acylated (*E. coli*, *P. aeruginosa*, *N. gonorrhoeae*), or even hepta‐ or octa‐acylated (*V. cholerae*). It can be further modified with groups such as phosphate‐ethanolamine (pEtN), 4‐amino‐4‐deoxy‐L‐arabinose (L‐Ara4N), or (di)glycyl moieties. In *H. pylori*, one sugar of Lipid A may also be dephosphorylated. Color scheme: Glucosamine I and its attached acyl chains are shown in *cyan*, Glucosamine II (linked to the sugar core) and its acyl chains in *magenta*, and additional substituent groups (pEtN, L‐Ara4N, di‐Glycyl) in *green*.

#### 2.1.2 SAAP‐148: Improved Activity With an Unexpected Twist

SAAP‐148 (Synthetic Antimicrobial and Anti‐biofilm Peptide 148) was designed by increasing the cationicity and helicity of the random coil C‐terminal region of LL‐37.^[^ ^65^ ^]^ In a study by Adélaïde et al.,^[^ ^66^ ^]^ both *in vitro* and *in silico* techniques were employed to assess SAAP‐148's activity against membrane models. The authors found that cholesterol and certain lipid head groups impaired SAAP‐148's membrane activity, preventing it from being toxic toward red blood cells (RBCs) or other mammalian cells.^[^ ^66^ ^]^ However, SAAP‐148 demonstrated increased interaction with bacterial membrane models, resulting in enhanced ordering of the acyl chains, likely contributing to its antimicrobial activity.^[^ ^66^ ^]^

The NMR and MD simulation study^[^ ^66^ ^]^ suggested a carpet‐like mechanism of SAAP‐148, consistent with observations that the helical‐prone region of LL‐37 (the LL‐32 peptide) binds to membrane surfaces without forming toroidal pores.^[^ ^62^ ^]^ Interestingly, the improved activity of SAAP‐148 compared to LL‐37 was achieved through a serendipitous change in its mechanism of action, illustrating that increasing antimicrobial efficacy does not necessarily follow a linear path.

#### 2.1.3 BMAP27 (Cathelicidin 6): Different Membranes, Different Mechanisms of Action

BMAP27, a bovine cathelicidin‐derived peptide, was studied by Sahoo et al.^[^ ^67^ ^]^ using all‐atom (AA)‐MD, using AMBER and CHARMM36 force fields together with coarse‐grained (CG)MD employing the Martini force field, which has recently been further optimized for studying protein‐membrane interactions.^[^ ^20^, ^68^ ^]^ The AA‐MD study revealed that BMAP27 loses helicity when interacting with POPC membranes but not with POPG membranes, a finding that mirrors the behavior of LL‐37.^[^ ^44^, ^58^, ^59^ ^]^ The authors suggested that BMAP27 acts via a carpet‐like mechanism in zwitterionic membranes (POPC) but forms toroidal pores in anionic membranes (POPG), similar to other helical‐kink peptides like LL‐37.^[^ ^69^ ^]^

This highlights how the mechanism of action responsible for the bactericidal activity may be very different from the one that causes toxicity in mammalian cells. The cytotoxic effect of BMAP27 was related to its hydrophobic C‐terminal domain, consistent with other studies showing how hydrophobicity and aggregation may promote promiscuous membrane activity.^[^ ^70^, ^71^ ^]^ This region promotes membrane insertion, in a manner reminiscent of the membrane‐penetrating properties of the bee venom toxin Melittin (Figure 3D). These findings underscore how membrane composition influences the mechanism of action of AMPs and highlights the importance of designing AMPs with selective activity to minimize toxicity toward mammalian cells.

#### 2.1.4 CM‐11: The Multifaceted Role of Tryptophan

CM‐11 is a synthetic chimeric peptide, composed of fragments from Cecropin A (positions 2‐8) and Melittin (positions 6‐9),^[^ ^72^ ^]^ that exhibits broad‐spectrum antimicrobial activity.^[^ ^73^, ^74^ ^]^ A recent MD study analyzed CM‐11's interaction with simplified models of Gram‐positive, (inner) Gram‐negative, and eukaryotic membranes.^[^ ^75^ ^]^ This study identified electrostatic interactions as the key driving force behind the binding of CM‐11 to the membrane, facilitated by the amphipathic nature of the peptide.^[^ ^75^ ^]^

Although not extensively discussed, snapshots of the simulations indicated that the N‐terminal tryptophan played a critical role in the early stages of membrane penetration. Tryptophan is a well‐known membrane‐penetrating residue, and its unique structural and physico‐chemical properties contribute significantly to membrane disruption in various AMPs.^[^ ^76^, ^77^ ^]^ Its membrane insertion depth is correlated with the overall hydrophobicity of the peptide, as demonstrated for Cecropin by Schlamadinger et al.,^[^ ^78^ ^]^ suggesting that Trp's positioning within the membrane is crucial for its function. Membrane surface destabilization was individuated as the mechanism of action also for the Legumin‐derived peptides *Leg1* and *Leg2*.^[^ ^79^ ^]^ *Leg2* shows a C‐terminal motif WLKL similar to the N‐terminal of both CM‐11 and CM‐15, and indeed the C‐ter residues of *Leg2* were confirmed to posses their energy minimum close to the membrane surface^[^ ^79^ ^]^ highlighting the role of Trp‐containing motifs to favorably interact with the phosphate‐headgroup region, even across unrelated peptides.

Cecropins, like CM‐11, are not highly hydrophobic and often remain near the membrane surface. This is due, in part, to the pyrrole NH group of the Trp indole ring, which forms hydrogen bonds with the phosphate groups of the lipids. As a result, Cecropins can promote multilamellar structure formation by interacting primarily with the surface of the membrane, rather than deeply penetrating it.^[^ ^80^, ^81^ ^]^

Additionally, the indole ring of Trp possesses a dipole moment that plays an important role in interactions with zwitterionic bilayers, such as those composed mainly of PE and PC lipids. Studies by Busath et al. have shown that increasing the dipole moment of the indole ring by using 5F‐indole can enhance the activity of the cyclic peptide Gramicidin A.^[^ ^82^ ^]^ In the context of Gramicidin, the hydrogen bonding capability of the indole ring facilitate the peptide's insertion into the phosphate region of the membrane, though this property may hinder deeper penetration into the hydrophobic core of the membrane.^[^ ^83^ ^]^

Surface‐binding, detergent‐like peptides such as Cecropins and CM‐11 benefit from these Trp‐mediated interactions at the membrane interface. However, for channel‐forming peptides, increased hydrophobicity might be desirable to promote deeper membrane insertion and pore formation. Various modifications of the Trp indole ring, such as methylation, tert‐butylation,^[^ ^84^ ^]^ and fluorination, have been proposed to modulate the hydrogen bonding ability, hydrophobic bulk and dipole moment of the indole ring. These modifications offer promising strategies for fine‐tuning the activity and selectivity of Trp‐containing peptides for specific membrane environments.

In conclusion, the multifaceted role of tryptophan in membrane‐active peptides like CM‐11 demonstrates the complex interplay between hydrogen bonding, hydrophobicity, and electrostatic interactions in determining the peptide's mechanism of action. Modifying Trp residues in AMP sequences offers a versatile approach to optimizing peptide activity for different target membranes.

#### 2.1.5 PGLa and Magainin 2: Cooperative Effects in Pore Formation

PGLa is a 21‐residue cationic amphipathic peptide found in the skin glands of *Xenopus laevis* frogs. It contains four cationic lysine residues, one polar serine residue, and sixteen hydrophobic residues.^[^ ^86^, ^87^ ^]^ PGLa exhibits hemolytic activity, with limited ability to differentiate between bacterial and mammalian membranes, as indicated by a therapeutic index (HC_50_/IC_50_) of less than 1 (i.e., its potency on human cells is comparable to that on bacterial cells).^[^ ^88^ ^]^ PGLa has been shown to form micelles^[^ ^89^, ^90^ ^]^ and undergo significant conformational changes when interacting with a DMPC/DMPG membrane mixture.^[^ ^90^, ^91^ ^]^ MD simulations by Bowers et al.^[^ ^91^ ^]^ using the same membrane model revealed that PGLa's helicity increases upon membrane binding, which is accompanied by lipid redistribution, causing DMPG lipids to cluster near the peptide. This mechanism (illustrated in Figure 3A) was initially hypothesized to explain the activity of some surface‐binding peptides.^[^ ^50^ ^]^

A follow‐up study by the same authors^[^ ^92^ ^]^ investigated PGLa dimerization, showing that PGLa has a low propensity for homodimerization. Instead, it preferentially forms heterodimers with Magainin‐2, another amphibian‐derived AMP.^[^ ^53^, ^93^ ^]^ Both experimental^[^ ^53^ ^]^ and computational^[^ ^93^ ^]^ studies agree that Magainin‐2 and PGLa have distinct roles in pore formation in the bacterial membrane: Magainin‐2 plays a stabilizing role in the pore structure, inserting shallowly into the membrane with a large tilt angle from the membrane normal, while PGLa inserts nearly parallel to the membrane normal, with a much smaller tilt angle (Figure 3C).^[^ ^93^, ^94^, ^95^ ^]^

This complementary behavior between Magainin‐2 and PGLa appears to be essential for their cooperative pore‐forming activity. Structural studies, including MD simulations and NMR experiments,^[^ ^91^, ^92^, ^93^, ^94^, ^95^ ^]^ indicate that PGLa can form toroidal pores, but only when associated with Magainin‐2. This interaction is mediated by the C‐terminal segments of both peptides. The association of two almost perfectly helical peptides via the slightly flexible terminal region of Magainin‐2 allows to form a dimer that mimics the behavior of helix‐kink‐helix peptides, known to induce toroidal pores.^[^ ^69^ ^]^ The cooperative pore formation highlights how synergistic interactions between different peptides can modulate their mechanisms of action.

The cooperative effects observed between PGLa and Magainin‐2 demonstrate the importance of peptide dimerization in determining their antimicrobial efficacy and, potentially, their toxicity. Different mechanisms of membrane penetration exhibit varying preferences for specific membrane compositions, making cooperativity an important consideration for optimizing the selectivity of AMPs.

Studying cooperative phenomena with MD simulations presents unique challenges, as larger systems and longer timescales are often required, which increases computational demands. However, enhanced sampling techniques allow for the high‐resolution study of systems and phenomena that are difficult to access with conventional simulations.^[^ ^96^ ^]^ Additionally, the synergy between NMR and MD techniques is critical: NMR can provide insights into molecular organization over relatively long timescales, while MD simulations enable the detailed exploration of molecular interactions and structural dynamics at very high spatial and temporal resolutions.

#### 2.1.6 Daptomycin: Lipid Extracting or Pore Forming Activity?

Daptomycin is a cyclic lipopeptide antibiotic produced by *Streptomyces roseosporus*, originally isolated from a soil sample collected at Mount Ararat, Turkey.^[^ ^5^ ^]^ Despite its more than 20 years of clinical use, Daptomycin resistance has been rare, though resistant strains of *Bacillus subtilis*, *Staphylococcus aureus*, and *Enterococcus faecium* are emerging.^[^ ^97^, ^98^ ^]^ Daptomycin is unusual in that it is an anionic peptide that targets bacterial membranes, which are also negatively charged. This specificity is facilitated by calcium ions (Ca^2 +)^), which promote Daptomycin oligomerization and mediate its interaction with bacterial cell membranes.

**Daptomycin and Calcium: How an anionic peptide targets an anionic membrane**. The mechanism of action of Daptomycin has been described as a *lipid‐extracting effect* by Chen et al.,^[^ ^99^ ^]^ with the decanoyl moiety at the N‐terminus playing a central role in this process. In addition to its lipid‐extracting activity, Daptomycin may also interact with bacterial membrane proteins, such as Usp2, leading to increased reactive oxygen species (ROS) production that results in bacterial cell death.^[^ ^100^ ^]^ The development of Daptomycin resistance has been associated with several factors, including cell wall thickening, alterations in membrane lipid composition, and changes in membrane fluidity.^[^ ^101^ ^]^ Karas et al. provided a comprehensive structure‐activity relationship (SAR) map for Daptomycin, highlighting the importance of the DxDG motif in forming a Ca^2 +^‐mediated complex that is fundamental for Daptomycin's activity,^[^ ^101^ ^]^ while other structural features are potentially modifiable to tweak the activity spectra and to improve the pharmacokinetic (PK) profile.

To further investigate the role of calcium in Daptomycin activity, Blasco et al.^[^ ^102^ ^]^ studied the differences in calcium chelation between Daptomycin and its close derivative Kynomycin (methylated on the Kynurenine residue).^[^ ^103^ ^]^ Ca^2 +^‐induced oligomerization is a fundamental step determining Daptomycin activity^[^ ^102^ ^]^ and derivatives with improved Ca^2 +^ chelation abilities may be more effective in terms of killing activity. The methyl‐Kynurenine, being more lipophilic than Kynurenine, tightly interacts with tryptophan and the lipid tail^[^ ^102^ ^]^ and these hydrophobic interactions improve the stability of oligomers. The possibility of exploring such subtle modifications (only one methyl group in this case) is an attractive path for the optimization of existing molecules.

Liu et al.^[^ ^104^ ^]^ used MD simulations to study Daptomycin's interaction with DMPC/DMPG (a bacterial membrane mimic) and pure DMPC (a mammalian membrane mimic). They found that Daptomycin oligomers were more stable in DMPC/DMPG mixtures than in pure DMPC, consistent with Daptomycin's selective activity against bacterial membranes. The simulations also revealed the formation of a narrow, water‐conducting channel within the Daptomycin tetramer, allowing ions to pass through and disrupt the membrane's potential in bacterial cells.^[^ ^104^ ^]^ This channel was stabilized by interactions between the carboxyl groups of Daptomycin and Ca^2 +^ ions, which shielded the hydrophilic moieties of the peptide from the membrane's hydrophobic core.

**Daptomycin multiple mechanisms of action: Controversy or synergy?** The literature on Daptomycin presents two possible mechanisms of action: the first involves detergent‐like extraction of lipids from the membrane,^[^ ^99^, ^102^ ^]^ while the second proposes the formation of a nanotube or pore spanning the bacterial membrane.^[^ ^104^ ^]^ These two mechanisms are likely not mutually exclusive. Nanotube formation could create destabilizing channels that lead to lipid extraction and the release of lipid vesicles, resulting in membrane damage.

While MD simulations have shown that the energy barrier for inserting a single Daptomycin molecule into the membrane is relatively high,^[^ ^104^ ^]^ cooperative effects and supra‐molecular complexes, involving both multiple Daptomycin molecules and calcium ions, can lower this energy barrier and facilitate membrane insertion. Daptomycin's ability to form oligomers or supra‐molecular complexes is widely supported by experimental evidence^[^ ^105^, ^106^, ^107^, ^108^ ^]^ and by MD simulation studies.^[^ ^102^, ^104^ ^]^ The improved activity of the Daptomycin derivative Kynomycin,^[^ ^103^ ^]^ which exhibits enhanced oligomerization, suggests that favoring oligomer formation may be an effective strategy for increasing Daptomycin's antibacterial potency.

#### 2.1.7 Lugdunin “the Fibupeptide”: The First of Many?

**Lugdunin: Ionophore mechanism of action**. Lugdunin is a cyclic peptide, isolated from a strain of nasal *Staphylococcus lugdunensis*.^[^ ^109^ ^]^ Its unique structure (depicted in **Figure** **4**A) alternates D‐ and L‐ amino acids and features a thiazolidine ring, a key component for Lugdunin's activity.^[^ ^110^ ^]^ This unusual configuration plays a pivotal role in how Lugdunin interacts with bacterial membranes. The peptide molecules form membrane pores, stabilized by hydrogen bonds between the amide nitrogen and oxygen atoms, creating an intermolecular β‐sheet‐like structure inside the membrane^[^ ^110^ ^]^ (Figure 4C).

MD simulations provided a detailed, atomistic view of Lugdunin's mechanism of action, showing that multiple peptide molecules assemble cooperatively to form a nanotube that spans the entire thickness of the bacterial membrane (Figure 4B,C). This nanotube remains stable on the microsecond timescale and has a diameter comparable to gramicidin and compatible with the passage of ions.^[^ ^110^ ^]^ MD simulations further revealed that Lugdunin rapidly inserts into the membranes of *S. aureus* and *B. subtilis*,^[^ ^110^ ^]^ with a slightly higher partitioning affinity for the *B. subtilis* membrane, a trend confirmed by experimental findings.^[^ ^111^ ^]^ Interestingly, while Lugdunin's neutral and lipophilic nature facilitates its membrane insertion, it is repelled by the negatively charged phosphates and Kdo (3‐Deoxy‐D‐manno‐oct‐2‐ulosonic acid) residues found in the lipopolysaccharide (LPS) of the outer membrane (OM) of Gram‐negative bacteria, such as *E. coli* (*unpublished data*). However, despite this repulsion, Lugdunin exhibited potent activity against *E. coli* with a compromised OM.^[^ ^111^ ^]^ The latter finding poses hopes for its use as a broad‐spectrum antibiotic in association with OM‐permeabilizing agents, an approach that is gaining attraction to re‐purpose Gram‐positive active molecules against the emergence of drug‐resistant Gram‐negative strains.^[^ ^112^ ^]^

The versatility of Lugdunin's ionophore‐like activity offers a compelling strategy for future antibiotic development. The ability of MD simulations to elucidate both the vertical aspects of its mechanism (e.g., membrane insertion, pore formation, and stabilization) and the horizontal aspects (e.g., structure‐activity relationships across different bacterial membranes) has proven invaluable. This mechanistic understanding was further supported by experimental techniques, such as Attenuated Total Reflectance‐Fourier Transform Infrared (ATR‐FTIR) spectroscopy and transmembrane current recordings, which confirmed the formation of stable nanotubes by Lugdunin and shed light its ion transport properties.^[^ ^110^ ^]^

**Lugdunin derivatives: From natural product to drug?** Editing of the structure of the Lugdunin scaffold confirmed the fundamental role of the thiazolidine moiety and of the alternating stereochemistry of the D‐ and L‐amino acids^[^ ^110^, ^113^ ^]^ and allowed to further explore Lugdunin SAR.^[^ ^114^ ^]^ An alanine scan of the peptide revealed the side chains that are amenable to synthetic alterations. The most active Lugdunin derivative was obtained by introducing a second tryptophan residue.^[^ ^113^ ^]^ Also, increasing the hydrophobicity of Lugdunin by substituting tryptophan with naphthalene‐ or anthracene‐alanine, resulted in active compounds.^[^ ^113^ ^]^ These modifications confirmed the importance of aromaticity and hydrophobicity in promoting membrane insertion, key factors that drive Lugdunin's mechanism of action.

In addition, a derivative of Lugdunin incorporating D‐propargylglycine, a modification that allows for future “click‐chemistry” modifications, broadens the potential scope of Lugdunin‐based therapies.^[^ ^113^ ^]^ The introduction of this modification opens up the possibility for further synthetic tailoring of the peptide, enabling the steered design of analogs with defined characteristics.

The promising selectivity and safety profile of Lugdunin and its derivatives suggest a high potential for development into a clinical drug product.^[^ ^109^, ^110^, ^113^ ^]^ Importantly, Lugdunin's structural characteristics fall within the chemical space of approved macrocyclic drugs, which bolsters the feasibility of advancing it toward clinical use.^[^ ^115^, ^116^, ^117^ ^]^ These advances render Lugdunin an exciting candidate in the ongoing battle against drug‐resistant bacterial infections, with the potential to inspire the discovery of other “fibupeptides” based on similar scaffolds.

#### 2.1.8 Polymyxins: The Founders of LPS Active AMPs

**Figure 11:**
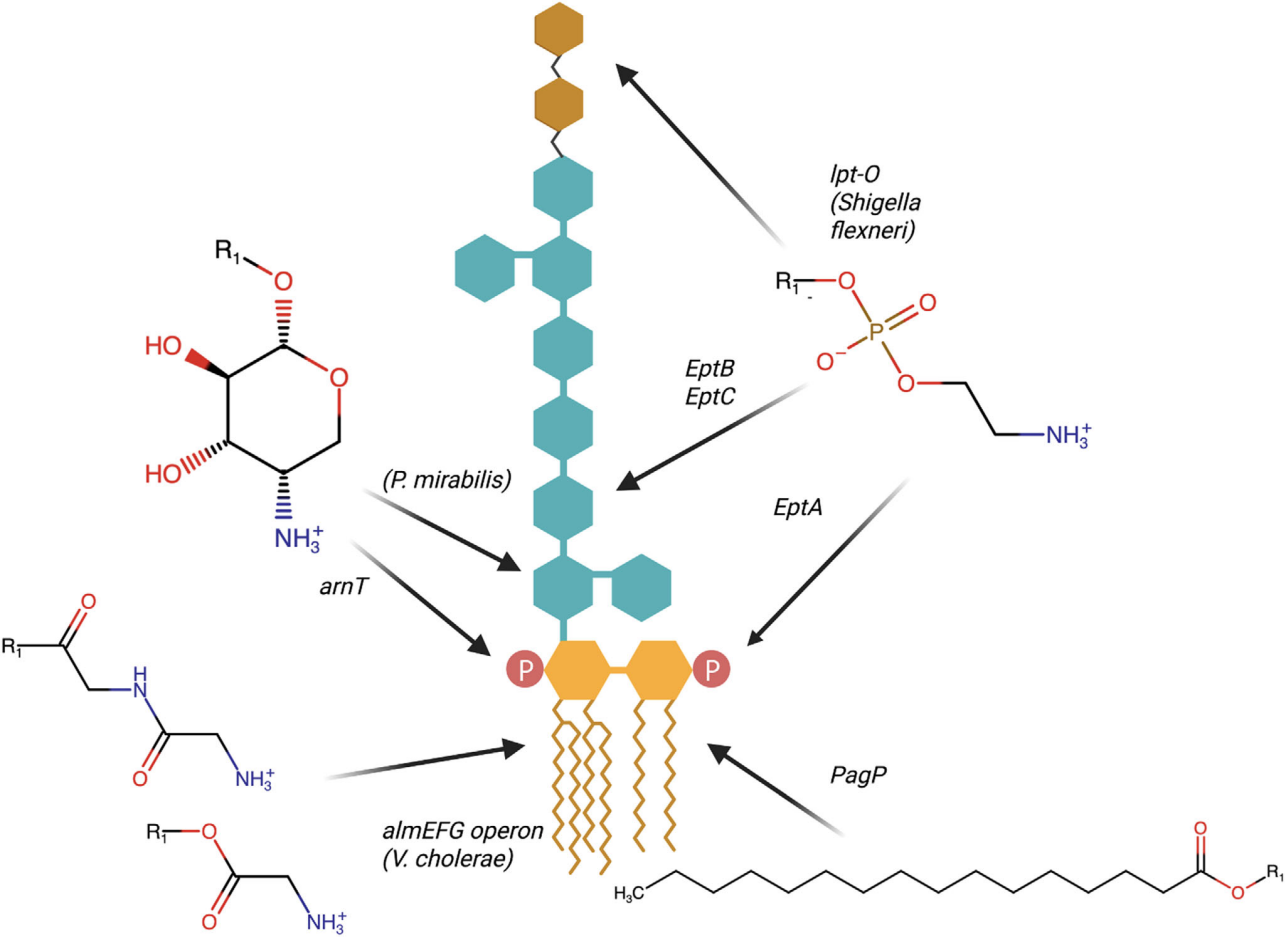
LPS modifications and their catalyzing enzymes. Certain Lipid A modifications are conserved across multiple Gram‐negative species, while others are species‐specific. The position labeled R1 indicates the attachment site. This schematic provides a general overview of the LPS structure, though the exact acylation pattern may vary slightly between species.

**Polymyxins: MD simulations as magnifying lenses over the LPS**. Polymyxins are poly‐cationic cyclic peptides produced by the soil bacterium *Paenibacillus polymyxa*,^[^ ^118^ ^]^ and they are known for their potent activity against Gram‐negative bacteria, exerting a membrane‐mediated mechanism of action.^[^ ^119^ ^]^ Polymyxins B and E (known as PMB and Colistin, respectively) have been widely investigated for their ability to interact with LPS molecules and to penetrate the OM)of Gram‐negative bacteria. PMB first binds to the LPS and then forms pores in the OM. The size of the PMB‐induced pores depends on the chemical decoration of the LPS, summarized in Figure 11. In the case of E. Coli or S. Enterica, the pores are large enough to allow self‐promoted uptake of PMB.^[^ ^120^ ^]^ In contrast, inclusion of 4‐amino‐4‐deoxy‐L‐arabinose at the Kdo of the LPS of P. mirabilis (highlighted in Figure 11) leads to a reduction in pore size, with consequent inhibition of the self‐uptake mechanism and resulting in strain resistance.^[^ ^121^ ^]^

MD simulations have played a crucial role in advancing our understanding of how polymyxins interact with LPS. A study by Li et al.^[^ ^122^ ^]^ demonstrated that the rigidity of the cyclic polymyxin scaffold is key for its activity by inducing deformations in the Lipid A portion of the LPS. Additionally, the introduction of phosphate‐attached ethanolamine (pEtN) groups contributes to polymyxin resistance by establishing strong salt bridges and hydrogen bonds with the phosphate of the adjacent Lipid A molecule and by further increasing the affinity of Lipid A for Ca^2 +^ ions by providing two closely tethered phosphate groups.^[^ ^122^ ^]^ These two phenomena result in the dehydration of the phosphate groups,^[^ ^122^ ^]^ rendering them less accessible to the positively charged groups of the polymyxins. The energetic penalty of breaking this intricate web of salt bridges and hydrogen bonds underpins the mechanism of mcr‐1‐mediated colistin resistance observed in Gram‐negative species of the ESKAPEE group.^[^ ^123^ ^]^

Another MD simulation study by Jiang et al.^[^ ^124^ ^]^ investigated the thermodynamics of polymyxin penetration into the bacterial membrane, employing a more realistic model of the Gram negative OM. Their energy profile revealed a shallow energy minimum at the interface between the Lipid A headgroup region and bulk water, with an energy maximum occurring deeper in the hydrophobic core of the membrane. This finding is consistent with the hydrophilic nature of both colistin and polymyxin B1. The study also suggested that polymyxins adopt a more folded conformation as they penetrate the hydrophobic region of the OM, aided by intramolecular hydrogen bonds.^[^ ^124^ ^]^ This folding behavior is characteristic of macrocyclic peptides, which are known for their “chameleonic” properties—that is, their ability to adapt their conformation to different environmental dielectric constants, a trait that is important for both PD and PK.^[^ ^115^, ^116^, ^124^, ^125^, ^126^ ^]^ The self‐promoted uptake of polymyxins is hence related to its peculiar macrocyclic structure and its cationic nature, capable of displacing the divalent cations Ca^2 +^ and Mg^2 +^, destabilizing the OM^[^ ^127^ ^]^

**Polymixins derivatives: Lower hydrophobicity, higher therapeutic index**. Polymyxins have been lifesaving drugs, particularly in treating multidrug‐resistant Gram‐negative infections. However, their clinical utility is hampered by significant safety concerns, particularly nephrotoxicity. Around 50–60% of patients receiving polymyxins experience dose‐limiting adverse effects, most commonly involving the kidneys.^[^ ^8^, ^10^ ^]^ Renal complications are especially dangerous in critically ill patients with comorbidities,^[^ ^10^ ^]^ underscoring the urgent need for polymyxin derivatives with improved safety profiles.

One promising derivative is F365 (QPX9003), which was designed inspired by polymyxin lipopeptides, but with key modifications that resulted in a significantly improved therapeutic index.^[^ ^128^ ^]^ The design of QPX9003 benefited from detailed structural models of the interaction between polymyxin B and LPS,^[^ ^119^, ^128^ ^]^ allowing to identify modification‐tolerant sites within the polymyxin scaffold. For example, the lipid tail of polymyxin was identified as a site that could be modified without compromising its essential binding interactions with LPS, as long as the hydrophobicity of the tail was maintained.

Modifications in other sites further reduced the overall lipophilicity (*logP*), which in turn decreased the interaction of the drug with lung surfactants and reduced its accumulation in the kidneys. These changes significantly improved the *in vivo* safety profile.^[^ ^128^ ^]^ Notably, the significant effects on toxicity caused by subtle stereochemical modifications and positioning of the halogen substituent on the benzyl ring of the new lipid tail^[^ ^128^ ^]^ highlighted how toxicity is not only influenced by physicochemical properties but likely involves other complex mechanisms (e.g., interactions with peptide transporters in the kidneys are likely important^[^ ^10^ ^]^).

In conclusion, polymyxins, as the founding members of LPS‐active AMPs, have set the stage for developing novel antibiotics targeting Gram‐negative bacteria. Advances in computational and experimental techniques continue to resolve the intricate interactions between polymyxins and bacterial membranes, revealing key insights into their mechanism of action and the development of resistance.

### 2.2 Inhibition of Cell Wall Synthesis: (Not) Another Brick in the wall

The bacterial cell wall is essential for maintaining the cell architecture and protecting against mechanical stress, making its synthesis pathways ideal targets for antimicrobial development. Inhibitors of cell wall synthesis impair the synthesis of peptidoglycan either by binding to enzymes or to precursors/substrates.^[^ ^129^, ^130^, ^131^, ^132^ ^]^ Both mechanisms block the peptidoglycan synthesis machinery, consequently weakening the cell wall and finally leading to cell lysis.^[^ ^132^, ^133^ ^]^

#### 2.2.1 From Vancomycin to Lipo‐Glycopeptides: Adding Weapons to the Armamentarium

Vancomycin, a non‐ribosomally synthesized peptide (NRP) in the glycopeptide antibiotic class, is one of the cornerstone drugs targeting cell wall synthesis, alongside other NRPs like polymyxins and gramicidins. Vancomycin's mechanism of action involves direct binding to the two D‐Ala residues at the C‐terminus of the pentapeptide region of Lipid II^[^ ^135^ ^]^ (shown in **Figure** **5**A,C), thereby impeding cell wall synthesis. Structurally, vancomycin is a glycopeptide, with a relatively polar structure and a large molecular weight, impermeable to the Gram negative OM.^[^ ^112^ ^]^

The main mechanism of resistance against vancomycin consists in the mutation of the terminal dipeptide in lipid II from D‐Ala‐D‐Ala to D‐Ala‐D‐Lac (shown in Figure 5C,D, respectively), and emerged in Vancomycin Resistant *Enterococcus* (VRE).^[^ ^136^ ^]^ This structural change eliminates one of the five hydrogen bonds between vancomycin and its target and increases the dissociation constant (*Kd*) by approximately 1,000‐fold.^[^ ^136^, ^137^, ^138^ ^]^ This small yet significant change underscores how minor structural alterations can drastically impact antibiotic potency.

To overcome vancomycin resistance both dual D‐Ala‐D‐Ala/D‐Ala‐D‐Lac binding derivatives,^[^ ^137^, ^139^ ^]^ and lipo‐glycopeptides derivatives have been synthesized.^[^ ^132^, ^140^, ^141^ ^]^ Lipo‐glycopeptides display a dual mechanism of action, blocking peptidoglycan synthesis and causing membrane depolarization.^[^ ^132^, ^140^, ^141^, ^142^, ^143^ ^]^ The membrane activity of lipo‐glycopeptides is conferred by a lipophilic tail attached to the typical glycopeptide scaffold.^[^ ^140^, ^141^, ^142^, ^143^ ^]^ The tail can be either aliphatic (Telavancin, Dalbavancin) or with aromatic rings (Oritavancin). This tail enables the lipo‐glycopeptides to anchor into the bacterial membrane, providing a concentrating effect that enhances both the disruption of peptidoglycan synthesis and membrane depolarization. The dual mechanism of action allows to overcome vancomycin resistance and makes lipo‐glycopeptides highly effective in clinical settings and less susceptible to the emergence of other resistance mechanisms.^[^ ^144^ ^]^

#### 2.2.2 Lantibiotics: Peculiar Amino Acids Confer a Peculiar Mode of Action

Lantibiotics represent another class of antimicrobial peptides that target lipid II, the critical precursor in bacterial cell wall synthesis, and display a dual functionality similar to lipo‐glycopeptides, combining cell wall inhibition with membrane‐disruptive action. Lantibiotics are ribosomally synthesized peptides characterized by the presence of lanthionine^[^ ^145^, ^146^ ^]^ (or its close relative, methyl‐lanthionine), as well as other non‐canonical unsaturated amino acids like dehydroalanine, and 2‐aminoisobutyric acid.^[^ ^145^ ^]^

Lanthionine is formed by cysteine and dehydrated serine, while methyl‐lanthionine derives from the coupling of cysteine and threonine.^[^ ^146^, ^147^ ^]^ The inclusion of lanthionine enables lantibiotics to form mono‐sulfide bridges, contributing to a partially cyclic, rigid macrocyclic structure that “cages” the pyrophosphate moiety of lipid II, as shown in Figure 5B.^[^ ^134^, ^148^, ^149^ ^]^ Lantibiotics may also have more than one mono‐sulfide bridge, that result in complex macrocyclic structures (e.g., Nisin in Figure 2 with the monosulfide bridges marked in blue).

The binding to lipid II inhibits cell wall synthesis, and serves as “landing terrain” for the subsequent formation of pores in the bacterial membrane.^[^ ^150^, ^151^, ^152^ ^]^ This two‐step mechanism of lantibiotics is confirmed by the induction of membrane depolarization exerted by lantibiotics like Nisin.^[^ ^153^ ^]^ The dual mechanism enables Nisin to target a broad range of Gram‐positive bacteria, although it is weakly active against Gram‐negative species due to the impermeability of the OM.^[^ ^154^ ^]^

Interestingly, under specific conditions, such as heat stress or exposure to EDTA (used in food preservation), the Gram‐negative OM is permeabilized. This allows Nisin to access and disrupt the IM and cell wall of Gram‐negative pathogens, such as *Salmonella* and *Escherichia coli* strains.^[^ ^86^, ^122^, ^154^, ^155^ ^]^ Moreover, recent developments in chimeric lantibiotic design have shown that combining Nisin with peptides capable of penetrating the OM can significantly enhance its effectiveness against high‐priority Gram‐negative pathogens like *A. baumannii* and *K. pneumoniae*.^[^ ^154^ ^]^

#### 2.2.3 Cell Wall Inhibitors: Moving Toward Broad Spectrum Activity?

Developing broad‐spectrum cell wall inhibitors has proven challenging, particularly for Gram‐negative bacteria, due to the protective OM that limits drug access to the cell wall. However, synergistic therapy strategies, in which two agents are used in combination — either as fixed‐dose or free‐dose regimens — offer a promising solution. By pairing a non‐OM permeable cell wall synthesis inhibitor (such as a glycopeptide or lantibiotic reviewed here) with a second compound that disrupts the OM, it may be possible to sensitize Gram‐negative bacteria to the action of non‐OM permeable cell wall synthesis inhibitors.^[^ ^112^ ^]^

This strategy opens new possibilities for expanding the application of glycopeptides and lantibiotics, both of which typically lack activity against Gram‐negative bacteria due to OM impermeability. Glycopeptides and lantibiotics exhibit dual mechanisms of action, combining cell wall synthesis inhibition with membrane‐disrupting properties, which not only increases their antibacterial efficacy but also reduces the likelihood of resistance development. When used in combination with OM‐permeabilizing agents, these bi‐functional antibiotics could potentially overcome barriers to Gram‐negative susceptibility.

The design of bi‐functional or chimeric molecules targeting both the OM and peptidoglycan synthesis, however, introduces significant challenges. Key among these is understanding the SAR for such complex agents. Multi‐scale modeling approaches are essential to elucidate how these drugs interact with various bacterial cell structures, including the OM, inner membrane (IM), lipid II, and the peptidoglycan layer. Through an integrated approach involving multi‐scale modeling, synergistic drug combinations, and SAR studies, a framework may be setup to systematically broaden the antimicrobial spectrum of (lipo‐)glycopeptides and lantibiotics toward Gram‐negative bacteria.

### 2.3 Targeting Intracellular Pathways and Proteins: A Look Inside the Cell

Many small‐molecule antibiotics achieve their antibacterial effects by targeting intracellular processes essential for bacterial survival. Clinically important key examples include quinolones and tetracyclines, which inhibit DNA and protein synthesis.^[^ ^156^, ^157^, ^158^ ^]^ Some AMPs, such as indolicidin and thiopeptides, also disrupt these intracellular pathways.^[^ ^159^ ^]^ This section discusses the mechanisms and therapeutic potential of these AMPs.

#### 2.3.1 Indolicidin and Derivatives: Structure Editing Enables Changes in the Activity Spectra

Indolicidin is a 13‐residue cationic AMP derived from bovine neutrophils, known for its broad‐spectrum antimicrobial activity and DNA‐targeting properties.^[^ ^161^, ^162^, ^163^ ^]^ Enriched in tryptophan and proline, it adopts a flexible random coil conformation both in solution and when bound to micelles (PDB IDs: 1g8c, 1g89)^[^ ^160^ ^]^ (**Figure** **6**A). Its amphipathicity, net positive charge, and C‐terminal amidation facilitate DNA binding through hydrogen bonding, salt bridges, and aromatic stacking interactions^[^ ^161^, ^162^ ^]^ (Figure 6B), which interfere with DNA biosynthesis.

The high tryptophan content promotes membrane association, as seen in micelle‐bound structures,^[^ ^160^ ^]^ enabling efficient cellular uptake and access to intracellular targets. Indolicidin exhibits a multi‐target or “dirty drug” profile, impacting various DNA biosynthesis enzymes.^[^ ^161^, ^162^ ^]^ This functional versatility makes it a compelling model forSAR studies, where targeted modifications can be employed to enhance selectivity against Gram‐positive or Gram‐negative pathogens.

Structure modifications have optimized indolicidin's activity and selectivity. Substituting three proline residues with alanine in the CP‐10 derivative enhances α‐helicity (structure deposited at PDB ID 1hr1) and shifts its action to membrane depolarization and destabilization, without losing its activity against intracellular targets^[^ ^164^ ^]^ (Figure 3A shows the mechanism of membrane activity, as described by Rozek et al.^[^ ^160^ ^]^). Despite this shift, CP‐10 retained its intracellular activity,^[^ ^164^ ^]^ likely due to enhanced membrane penetration, which increases the intracellular concentrations of the peptide.

To optimize indolicidin's efficacy against Gram‐negative bacteria, increasing the peptide cationicity was necessary, leading to the development of CP‐11 (PDB ID: 1qxq).^[^ ^165^, ^166^ ^]^ A macrocyclic variant, cyclo‐CP‐11^[^ ^166^ ^]^ (PDB ID: 1qx9), was synthesized to improve the peptide half‐life by reducing susceptibility to proteolytic degradation by trypsin enzymes, which commonly cleave basic, Arg‐rich peptides. Cyclo‐CP‐11 demonstrated prolonged stability in the presence of trypsin,^[^ ^166^ ^]^ illustrating how cyclization can enhance the pharmacokinetic properties of AMPs. Both CP‐11 and cyclo‐CP‐11 retained low minimum inhibitory concentrations (MICs) against Gram‐positive bacteria.^[^ ^166^ ^]^ CP‐11 peptide has a better hemolytic profile compared to indolicidin,^[^ ^166^ ^]^ confirming the hypothesis that a reduction in hydrophobicity may reduce hemolytic activity.^[^ ^70^, ^71^ ^]^ The researchers also observed that intracellular effects on the DNA biosynthesis pathway and downstream ones (RNA and protein synthesis) were still conserved in both CP‐11 and cyclo‐CP‐11.^[^ ^165^, ^166^ ^]^

These findings reveal that significant modifications to indolicidin's amino acid sequence and 3D structure do not impair its intracellular activity. Instead, these modifications highlight the broad and adaptable mechanism of action of the peptide, as its interactions with DNA and associated biosynthetic enzymes do not rely on highly specific molecular recognition but rather involve more promiscuous interactions. Studies by Rozek et al.,^[^ ^160^, ^166^ ^]^ Friederich et al.,^[^ ^164^ ^]^ and Falla et al.^[^ ^165^ ^]^ demonstrate how natural peptide sequences can be tailored in multiple ways to enhance one or more mechanisms of action, effectively shifting indolicidin's activity spectrum to target specific bacterial species.

#### 2.3.2 Thiopeptides: Balancing Activity and Solubility

Thiopeptides are a diverse class of ribosomally synthesized and post‐translationally modified peptides (RiPPs), secreted by Gram‐positive bacteria such as *Firmicutes* and *Actinomyces*. These bacteria use thiopeptides to compete against other Gram‐positive species, including *Clostridium difficile*, *Staphylococcus aureus*, *Streptococcus pneumoniae*, and *Mycobacterium tuberculosis*.^[^ ^167^, ^168^ ^]^ Due to their high potency in inhibiting bacterial protein synthesis,^[^ ^167^, ^168^, ^169^ ^]^ thiopeptides are promising candidates for novel antibiotic development. For example, Novartis developed the thiopeptide derivative LFF571A by enhancing the solubility and chemical stability of the natural product GE2270,^[^ ^170^, ^171^, ^172^ ^]^ a known potent inhibitor of the prokaryotic elongation factor thermo unstable (EFTu).^[^ ^173^ ^]^ EFTu catalyzes the binding of aminoacyl‐tRNA (aa‐tRNA) to ribosomes,^[^ ^174^ ^]^ an essential step in proteintranslation.

Following similar principles, Kim et al.^[^ ^175^ ^]^ designed novel AMPs based on Micrococcin 2, a thiopeptide with activity against *Clostridium difficile*.^[^ ^176^, ^177^ ^]^ Micrococcin 2 binds tightly to a cleft between the 23S rRNA subunit and the L11 C‐terminal domain (CTD), inhibiting the binding of elongation factor G (EF‐G).^[^ ^178^ ^]^ EF‐G binding basically allows the ribosomes to continue “reading” the mRNA in order to translate it to the protein sequence. Kim et al.^[^ ^175^ ^]^ aimed to increase Micrococcin 2's solubility by adding highly polar groups to the scaffold, limiting its gastrointestinal absorption. The latter is fundamental to confine the drug in the intestinal lumen (where *C. difficile* resides and produces its toxins) while reducing the systemic exposure to the drug.^[^ ^172^, ^179^ ^]^ These structural modifications were guided by docking generated poses and MM/GBSA (Molecular Mechanics/Generalized Born Surface Area) calculations to assess the binding energy,^[^ ^175^ ^]^ underscoring the importance of structure‐based design in optimizing compound properties.

Thiopeptides hold considerable clinical potential,^[^ ^180^, ^181^ ^]^ as evidenced by Thiostrepton's use in veterinary medicine as a topical ointment. However, poor solubility has restricted their application in human‐use drugs.^[^ ^168^, ^182^ ^]^ Thiopeptides are attractive scaffolds to develop novel antibiotics thanks to their potent activity against key cellular processes. Thiopeptides bearing a 26‐membered macrocyclic core (e.g., Thiostrepton, Micrococcin peptides) bind the ribosomal protein L11 and the 23S rRNA with high affinity^[^ ^183^ ^]^(Thiostrepton‐bound structure shown in Figure 6C), effectively blocking the binding of the elongation factor EF‐G.^[^ ^184^, ^185^, ^186^, ^187^ ^]^ Notably, these thiopeptides act on highly conserved bacterial structures^[^ ^188^ ^]^ while sparing eukaryotic ribosomes,^[^ ^189^ ^]^ minimizing toxicity. Some thiopeptides, including GE2270 and other 29‐membered macrocycles, act by inhibiting the ternary complex formation of EFTu‐GTP‐tRNA, leading to similar effects on bacterial protein synthesis.^[^ ^168^, ^169^ ^]^

While thiopeptides exhibit strong bactericidal activity and selectivity, their low solubility remains a barrier to clinical use. Research into synthetic routes for thiopeptide modifications is ongoing,^[^ ^190^, ^191^, ^192^ ^]^ and poses great potential for translating these compounds into the clinic. Notably, recent findings show that some thiopeptides can permeate the outer membrane of certain Gram‐negative species via species‐specific porin proteins,^[^ ^182^ ^]^ highlighting their potential for developing broad‐spectrum antibiotics. Further work is needed to provide a detailed SAR of thiopeptides and to explore bioisosteric replacements that can improve their solubility and expand the therapeutic scope of thiopeptides.

### 2.4 Targeting Biofilm Formation: Peptides put the Bacterial Fortress Under Siege

A biofilm is defined as an aggregate of microorganisms embedded in a self‐produced extracellular polymeric substance (EPS).^[^ ^193^ ^]^ Biofilm formation typically enhances the antibiotic resistance of bacteria compared to their free‐floating (planktonic) counterparts.^[^ ^194^, ^195^, ^196^ ^]^ The EPS serves as an additional barrier that restricts antibiotic permeation, while the metabolic activity of bacteria within a biofilm often differs significantly from that of planktonic cells.^[^ ^194^, ^195^, ^197^, ^198^ ^]^ Biofilms also protect bacteria from detergents and surface disinfectants,^[^ ^199^, ^200^, ^201^, ^202^ ^]^ making biofilm formation especially problematic in healthcare environments where high selection pressure often drives the evolution of antibiotic‐resistant strains.^[^ ^203^, ^204^, ^205^, ^206^, ^207^ ^]^ Biofilms are equally challenging for implants,^[^ ^208^, ^209^, ^210^, ^211^ ^]^ which represent an attractive application for surface‐coating AMPs.^[^ ^212^ ^]^

Before discussing the mechanisms by which peptides target biofilms, it is important to distinguish between Anti‐biofilm Peptides (ABPs) and Biofilm Inhibiting Peptides (BIPs), as well as the measures used to assess their effectiveness: Minimum Biofilm Inhibitory Concentration (MBIC) and Minimum Biofilm Eradication Concentration (MBEC).^[^ ^213^, ^214^ ^]^ MBIC indicates a peptide's efficacy in preventing biofilm formation,^[^ ^215^ ^]^ largely independent of its ability to penetrate the extracellular matrix, making it a suitable metric for BIPs. Conversely, MBEC evaluates a peptide's efficacy against pre‐formed biofilms,^[^ ^215^ ^]^ where activity likely depends on the peptide's capacity to reach bacterial cells within the dense ECM. MBEC is thus the appropriate measure for ABPs.

In this section, we will focus on ABPs to explore how the unique conditions within the biofilm environment have influenced the evolution of ABP sequences and their mechanisms of action.

#### 2.4.1 How ABPs Differ from Other AMPs? Insights from Machine Learning

To understand how the biofilm environment influences peptide structures and to inform the design of novel ABPs, Bose et al.^[^ ^214^ ^]^ applied a ML algorithm to identify the characteristic features of ABPs. The study revealed that ABPs, i.e., peptides active against pre‐formed biofilms, are typically short (most below 20 residues), cationic, amphipathic, and predominantly structured in either α‐helical or β‐stranded conformations.^[^ ^214^ ^]^ ABPs were also found to contain higher proportions of Arginine, Lysine, and Tryptophan residues.^[^ ^214^ ^]^

Arginine binds with its bidentate guanidine group tightly to phosphate groups and induces membrane thinning.^[^ ^216^, ^217^ ^]^ The side chain length of arginine enables interaction with phosphates in the distal leaflet of the membrane, especially when a pore opens, and the specific distance between the backbone and the guanidine group is a fundamental characteristic to promote cellular uptake.^[^ ^218^ ^]^ Lysine, though less efficient in translocation compared to Arginine,^[^ ^218^ ^]^ can “snorkel”^[^ ^94^ ^]^ to stabilize a pore, a property it shares with arginine.^[^ ^219^, ^220^ ^]^ Tryptophan is well known for its membrane‐insertion capabilities, which support effective peptide anchoring.^[^ ^76^, ^77^, ^221^, ^222^ ^]^

These characteristics suggest that membrane‐targeting mechanisms are likely predominant among ABPs. Additionally, the observation that ABPs are shorter and typically more structured compared to AMPs may suggest that extracellular matrix selects for more compact structures, while larger and more flexible conformations can get stuck in this dense web of polymers.

#### 2.4.2 Challenges in the Study of ABPs Activity

The dense EPS complicates the study of ABPs and their mechanisms of action. “Peptide 1018” provides an illustrative example. De la Fuente‐Núñez et al. studied peptide 1018, noting moderate potency against planktonic *P. aeruginosa* but high activity against pre‐formed biofilm populations.^[^ ^223^ ^]^ This led the authors to hypothesize that peptide 1018 targets a molecular mechanism unique to the biofilm microenvironment, possibly related to stress response pathways.^[^ ^223^ ^]^ However, Andresen et al. later disproved this hypothesis.^[^ ^224^ ^]^ The initial MBIC/MBEC values were found to be inaccurate, potentially due to the accumulation of the cationic peptide in the biofilm within flow cell chambers, resulting in a local peptide concentration higher than anticipated.^[^ ^224^ ^]^ Although peptide 1018 was confirmed to bind to its proposed target, ppGpp, the target was not essential for its antimicrobial action.^[^ ^224^ ^]^ This finding highlights the promiscuity and accumulation tendencies of certain cationic peptides in biofilms, where anionic polymers like alginic acid are abundant.^[^ ^198^ ^]^ On the other hand, this phenomenon presents an opportunity to explore the factors that drive peptide accumulation within biofilms, potentially revealing strategies to enhance ABP potency against those ecological niches.

ABPs remain a compelling area of research, particularly given the urgent need for solutions to counteract biofilm‐related resistance. Evolution has naturally selected some of the critical features of effective ABPs, such as their compact structure. Highly potent ABPs have promising applications in the surface‐coating of prosthetic and medical devices, where bioavailability is less critical. The biofilm environment, with its dormant cells and intricate cell‐to‐cell interactions, offers a unique context for discovering or designing peptides with novel mechanisms of action, such as reactivating dormant synthetic pathways to re‐sensitize bacteria toward cell wall, protein, or DNA synthesis inhibitors. The use of (highly) coarse‐grained molecular modeling approaches to study ABP interactions with ECM polymers may also provide structural insights that align with experimental findings.

## 3 Composition, Structure, and Dynamics of Bacterial Membranes Versus Eukaryotic Membranes

### 3.1 Gram‐Positive Bacteria

The peptidoglycan layer in Gram‐positive bacteria presents relatively large pores, which allow peptides and small organic molecules to access the outer side of the cell membrane with relative ease.^[^ ^225^, ^226^ ^]^ This peptidoglycan forms a protective shell around the Gram‐positive cell, with a thickness ranging between 30 nm and 100 nm.^[^ ^227^ ^]^ In general, Gram‐positive bacteria possess a thicker peptidoglycan cell wall compared to the thinner peptidoglycan layer of Gram‐negative bacteria within their periplasm.^[^ ^227^, ^228^ ^]^ The cell wall of Gram‐positive bacteria is rich in charged and polar molecules and may be modified by wall teichoic acids (WTAs) or serve as a scaffold for non‐covalent interaction of proteins. Both functions contribute to certain antibiotic resistance mechanisms by altering the physicochemical and structural properties of the wall, thus enhancing its ability to trap molecules.^[^ ^229^, ^230^ ^]^ The primary role of the peptidoglycan layer is to sustain the osmotic pressure and turgor of the cell,^[^ ^227^ ^]^ maintaining cell structure. However, it has limited capacity to impede the penetration of xenobiotics, a role primarily carried out by the cell membrane.^[^ ^231^ ^]^

The Gram‐positive bacterial membrane is composed of a variety of phospholipids, glycolipids, and other molecules such as menaquinone:^[^ ^232^ ^]^

- Phosphatidylglycerol
- Phosphatidylethanolamine
- Cardiolipin
- Glycolipids
- Menaquinone

The Gram‐positive cell membrane is an asymmetric bilayer composed of phospholipids, cardiolipins, and glycolipids (structure model in **Figure** 7C, percentage membrane compostion in **Figure** 8).^[^ ^233^, ^234^, ^235^ ^]^ The proportions of these components can vary significantly across different species and throughout the bacterial life cycle.^[^ ^236^, ^237^ ^]^ The membrane composition is highly adaptable and influenced by environmental factors such as pH, temperature, osmotic pressure, nutrient availability, and antibiotic exposure.^[^ ^98^, ^236^, ^237^, ^238^, ^239^, ^240^ ^]^

**Phosphatidylglycerol (PG)** is the most abundant phospholipid in Gram‐positive cell membranes.^[^ ^241^, ^242^ ^]^ PG can exist in its native form or be esterified to an amino acid, typically alanine or lysine (**Figure** 9).^[^ ^238^, ^241^, ^242^, ^243^ ^]^ It is a negatively charged phospholipid with two acyl chains (typically 16–18 carbons in length) and a headgroup characterized by two hydroxyl groups and the anionic phosphate group.^[^ ^244^, ^245^ ^]^ PG interacts with cations,^[^ ^246^ ^]^ particularly divalent ones like calcium,^[^ ^154^, ^247^ ^]^ which can bridge negatively charged membrane components and facilitate bacterial aggregation by neutralizing the cell surface charge.^[^ ^248^, ^249^ ^]^ Calcium presence also plays a role in the efficacy of AMPs such as daptomycin.^[^ ^104^ ^]^ The modification of PG with alanyl‐ or lysyl‐groups increases the amount of positive charges at the surface (alanyl‐PG is zwitterionic, lysyl‐PG carries a +1 net charge). For example, in *S. aureus*, lysyl‐PG is more abundant during the logarithmic growth phase,^[^ ^250^ ^]^ increasing resistance to cationic AMPs by the increased surface charge and by promoting tighter membrane packing.^[^ ^242^ ^]^

**Phosphatidylethanolamine (PE)** is a zwitterionic phospholipid featuring a positively charged ethanolamine phosphoesterified to a negatively charged phosphate group (Figure 9). The acyl chains of PE lipids in Gram‐positive membranes are typically 16‐18 carbon atoms in length, which may be fully saturated, (usually mono‐) unsaturated, or cyclopropanated.^[^ ^233^, ^251^ ^]^ Cyclopropanation, which increases under stress or stationary growth phases at the expense of unsaturated acyl chains,^[^ ^252^ ^]^ enhances membrane rigidity, thus reducing the permeability to AMPs and small‐molecule antibiotics. The function of PE lipids in the membrane and in its interaction with AMPs is also related to its ability to accumulate in high‐curvature regions. The high negative curvature tendency of PE is due to its inverted conical shape, favoring its localization in the septal and polar regions of the cell, with a distribution partially similar to that of cardiolipins.^[^ ^253^ ^]^

**Cardiolipin (CL)** is a tetra‐acylated phospholipid with two (at neutral pH negatively chrged) phosphate groups and four acyl tails, generally 16–18 carbons in length and possibly saturated, mono‐unsaturated, cyclopropanated, or branched (either in the iso‐ anteiso‐ conformation).^[^ ^244^, ^254^ ^]^ CL's characteristic inverted cone shape and the associated negative spontaneous curvature leads to its accumulation in highly curved membrane domains, such as the septal and polar ones.^[^ ^253^, ^255^ ^]^ This accumulation of PE and CL in highly curved regions is common to both Gram‐positive and Gram‐negative cell membranes. The level of CL is higher in species like *Staphylococcus aureus*, especially in the L‐form (lacking cell wall), suggesting that CL may interact with cations to stabilize the membrane when the cell is more vulnerable.^[^ ^241^ ^]^ Such membrane stabilizing role may have important consequences for AMPs susceptibility.

**Glycolipids** play a crucial role in AMP interactions due to their large, polar headgroups (Figure 9. Bacterial glycolipids include mono‐, di‐, tri‐, and tetra‐glucosyl‐diglycerides, as well as glycero‐phospho‐diglucosyl‐diacylglycerols (GPDGDAG).^[^ ^256^ ^]^ Glycolipids are essential for cell morphology and biofilm formation.^[^ ^257^, ^258^ ^]^ Their abundance varies, with species such as *Streptococcus pneumoniae* and *Enterococcus faecalis* exhibiting high levels of glycolipids (>50%) like di‐glucosyl diacylglycerols and GPDGDAG.^[^ ^259^, ^260^ ^]^ GPDGDAG also cooperates with lysyl‐PG in the development of Daptomycin resistance in *Enterococcus faecium* strains^[^ ^260^, ^261^ ^]^ by forming interactions between the phosphate of GPDGDAG and lysyl‐PG amino groups. Moreover, GPDGDAG is an important precursor of WTAs,^[^ ^261^ ^]^ highlighting the role of glycolipids as an important linking element between the Gram‐positive membrane and the cell wall.

**Menaquinones (MK)** are the main isoprenoid quinones found in both Gram‐positive and Gram‐negative bacteria, as well as in mycobacteria.^[^ ^232^, ^262^ ^]^ MKs are characterized by a naphthoquinone head and a variably repeated isoprene unit as tail (typically 7–9 units).^[^ ^263^ ^]^ In many Gram‐positive species the double bond in the β‐isoprene unit is reduced, causing the partial saturation of the isoprenyl side chain.^[^ ^264^ ^]^ The predominant, reduced form of MK in *Mycobacterium tuberculosis* enhances electron transport efficiency.^[^ ^264^ ^]^ The enzyme MenJ, which mediates MK reduction, is essential for survival within host macrophages, making it a promising antibiotic target.^[^ ^264^ ^]^ Although MK is present in very small quantities (around 1 %),^[^ ^232^ ^]^ its peculiar structure makes it an attractive target for a class of AMPs defined as Menaquinone‐Binding Antibiotics (MBAs), among which Lysocins are well represented.^[^ ^265^, ^266^, ^267^ ^]^ Lysocins are a class of AMPs produced by the Gram‐negative bacteria *Lysobacter* ^[^ ^268^ ^]^ that possess the ability to damage bacterial membranes containing MKs.^[^ ^265^, ^266^ ^]^

**Phosphatidylcholine (PC)**, a zwitterionic phospholipid, comprises a choline moiety (positively charged), a phosphate group, the glycerol moiety, and two acyl chains. Lyso‐Phosphatidylcholines (lyso‐PCs) derive from PC lipids by removal of an acyl chain.^[^ ^269^ ^]^ Although PC and lyso‐PCs are rare in bacteria, some Gram‐positive species like *S. pneumoniae*, *S. pyogenes* and *S. agalactiae* can scavenge PC‐derived metabolites, to feed the glycerophosphocholine (GPC) pathway and synthesize PC lipids.^[^ ^270^, ^271^, ^272^ ^]^ Joyce et al. hypothesized that PC lipids may help bacteria evade immune recognition, thus modulating host‐pathogen interactions.^[^ ^270^ ^]^ Furthermore, plasmalogens can function as antioxidants, and thanks to their highly unsaturated tails, they can potentially aid bacterial survival in oxidative environments such as during phagocytosis.^[^ ^273^ ^]^

In summary, Gram‐positive membranes contain variable amounts of PG, PE, CL, glycolipids, menaquinones, and occasionally PC and plasmalogens. PG is overrepresented in the outer leaflet, particularly in its amino‐acylated forms, which play a key role in AMP interactions. PE and CL localize to curved regions of the inner leaflet. Glycolipids are mainly antennae toward the external environment and accordingly preferentially within the outer leaflet, although in such species their abundance is so high that they are likely significantly present in the inner leaflet as well. MK, due to its high hydrophobicity, may flip between leaflets, possibly positioning also perpendicular to the membrane normal. PC lipids, implicated in immune evasion, reside mainly in the outer leaflet.

### 3.2 Gram‐Negative Bacteria

Gram‐negative bacteria are particularly challenging due to their high propensity for developing resistance mechanisms. Notably, five out of the seven ESKAPEE pathogens are Gram‐negative, including *Klebsiella pneumoniae*, *Acinetobacter baumannii*, *Pseudomonas aeruginosa*, *Enterobacter spp*., and *Escherichia coli*, with *E. coli* ranking first in deaths associated with antibiotic resistance.^[^ ^274^ ^]^

The intrinsic resistance of Gram‐negative bacteria largely stems from their highly impermeable OM .^[^ ^275^ ^]^ The OM is a highly asymmetrical bilayer, consisting of LPS in the outer leaflet and a combination of phospholipids — mainly PE and PG — and varying levels of CLs in the inner leaflet (structure models shown in Figure 7 A and B, percentage composition in Figure 8).^[^ ^227^ ^]^ Although the bilayer's hydrophobic thickness is similar to that of typical phospholipid bilayers, other physicochemical properties are significantly distinct.^[^ ^276^ ^]^ The dense layer of sugar residues in the LPS serves as a unique steric and electrostatic barrier against antibiotic penetration, affecting bilayer dynamics and porin functionality.

LPS sugar chains vary widely in length and composition. They include an inner core (typically composed of Kdo and heptose sugars), a species‐specific outer core, followed by a highly variable O‐antigen. Hence, variability increases outward, with the inner components (e.g., the Lipid A disaccharide) more conserved and the outer O‐antigen being the most variable region, even within the same species.^[^ ^277^ ^]^ This diversity is relevant for the bacterial serotype determination and in particular for interactions with the immune system. The core sugars attached to LPS also act as a first barrier to the permeation of antibiotics.^[^ ^278^ ^]^ Lipid A, the bilayer‐forming component of LPS, is relatively conserved but still modifiable through enzymatic modifications to alter immunogenicity and membrane properties. **Figure** **10** illustrates how Lipid A can be altered through modifications at the level of the phosphate groups with either a small zwitterion or a sugar substituent, and variations in acyl chain numbers, impacting AMP resistance and the innate immune response. The considerable structural variability across all three LPS components, enabled by various enzymes, allows Gram‐negative bacteria to adapt their outer membrane properties in response to environmental stresses, including antibiotic treatment.^[^ ^122^, ^123^, ^124^, ^279^, ^280^, ^281^ ^]^

The robust barrier of the OM is complemented by a seemingly thinner inner membrane that nonetheless poses significant resistance to antibiotic permeation and is a critical target for many AMPs. The IM of Gram‐negative bacteria resembles that of Gram‐positive species, comprising mainly PE, PG, and CL. However, in Gram‐negative bacteria, PE is the predominant lipid, followed by PG, with CL comprising approximately 5% and concentrated in polar and septal regions.^[^ ^282^, ^283^, ^284^ ^]^ Unlike Gram‐positive bacteria with a significant number of aminoacylated PG, PG lipids in Gram‐negative species are mostly unmodified. Gram‐negative species also lack iso‐ and anteiso‐branched acyl chains, though cyclopropanated and unsaturated chains are abundant. The PE:PG ratio in *E. coli* is around 4, with a higher ratio in the periplasmic leaflet and a lower one in the cytosolic leaflet.^[^ ^236^, ^252^ ^]^ This composition indicates a greater abundance of PE in the Gram‐negativeIM compared to the Gram‐positive bacterial membrane. The preferential cytosolic orientation of PG lipids aligns with the “positive‐inside” rule, where positively charged cytosolic loops of transmembrane proteins preferentially interact with PG or CL.

As with Gram‐positive bacteria, acyl chain composition in Gram‐negative membranes is dynamic, changing throughout the life cycle. Cyclopropane modifications increase from early log to stationary phases, enhancing resistance to environmental stresses.^[^ ^285^, ^286^, ^287^ ^]^ This increase in cyclopropanated chains is accompanied by a decrease in unsaturated tails,^[^ ^252^ ^]^ leading to higher lipid ordering in the early log phase and a progressive membrane thinning during the stationary phase.

#### 3.2.1 LPS Modifications: AMPs Resistance and Immunity

LPS is a highly variable molecule, with modifications to Lipid A, core sugars, and O‐antigen enabling bacterial cells to adapt to environmental stresses, evade immunity, and resist antibiotics.^[^ ^288^ ^]^ These modifications are critical in conferring resistance also to endogenous AMPs while modulating the activation of Toll‐like receptor 4 (TLR4, also CD284). Gram‐negative bacteria suppress TLR4 activation by altering Lipid A structure to delay or evade innate immune responses while reinforcing LPS‐LPS interactions with cationic moieties to prevent AMP activity.

*H. pylori* provides a prime example of this dual strategy, thriving in the harsh gastric mucosa environment. To survive, *H. pylori* extensively modifies its LPS: phosphorylation and the O‐antigen sequence are optimized for survival in the stomach, Lipid A acylation is reduced from six to four chains, thereby reducing TLR4 activation,^[^ ^279^, ^289^ ^]^ mimicking the TLR4 antagonist Eritoran (TLR4‐Eritoran complex at PDB ID 2z65^[^ ^290^ ^]^). As illustrated in Figure 10 the number of acyl chains induces different conformational changes in TLR4,^[^ ^290^ ^]^ resulting in a positive correlation between the number of acyl chains in Lipid A and TLR4 activation.^[^ ^291^ ^]^ These changes also neutralize Lipid A's phosphate groups via pEtN addition (by EptA)^[^ ^292^ ^]^ and phosphate removal (by LpxG), rendering the outer membrane highly resistant to cationic AMPs^[^ ^279^ ^]^ and antibiotics like polymyxins.^[^ ^292^ ^]^ The phosphate removal, in particular, significantly raises polymyxin B's minimum inhibitory concentration (MIC), a decisive feature for host colonization.^[^ ^292^ ^]^ Furthermore, *H. pylori* expresses Lewis O‐antigens (sugar sequences mimicking blood groups antigens), sugar sequences mimicking human blood group antigens, enabling immune evasion.^[^ ^279^, ^293^ ^]^

Other species employ similar strategies. pEtN or ppEtN modifications to Lipid A and inner core Heptose sugars stabilize LPS‐LPS and Ca^2 +^‐LPS interactions, forming a dense steric and electrostatic barrier against AMPs.^[^ ^123^, ^279^, ^292^, ^294^ ^]^ These modifications render LPS‐AMP interactions energetically unfavorable, further enhancing resistance. Divalent cations, essential for LPS stability under normal conditions,^[^ ^295^ ^]^ synergize with pEtN/ppEtN modifications to strengthen the LPS‐LPS interactions.

**Figure 12:**
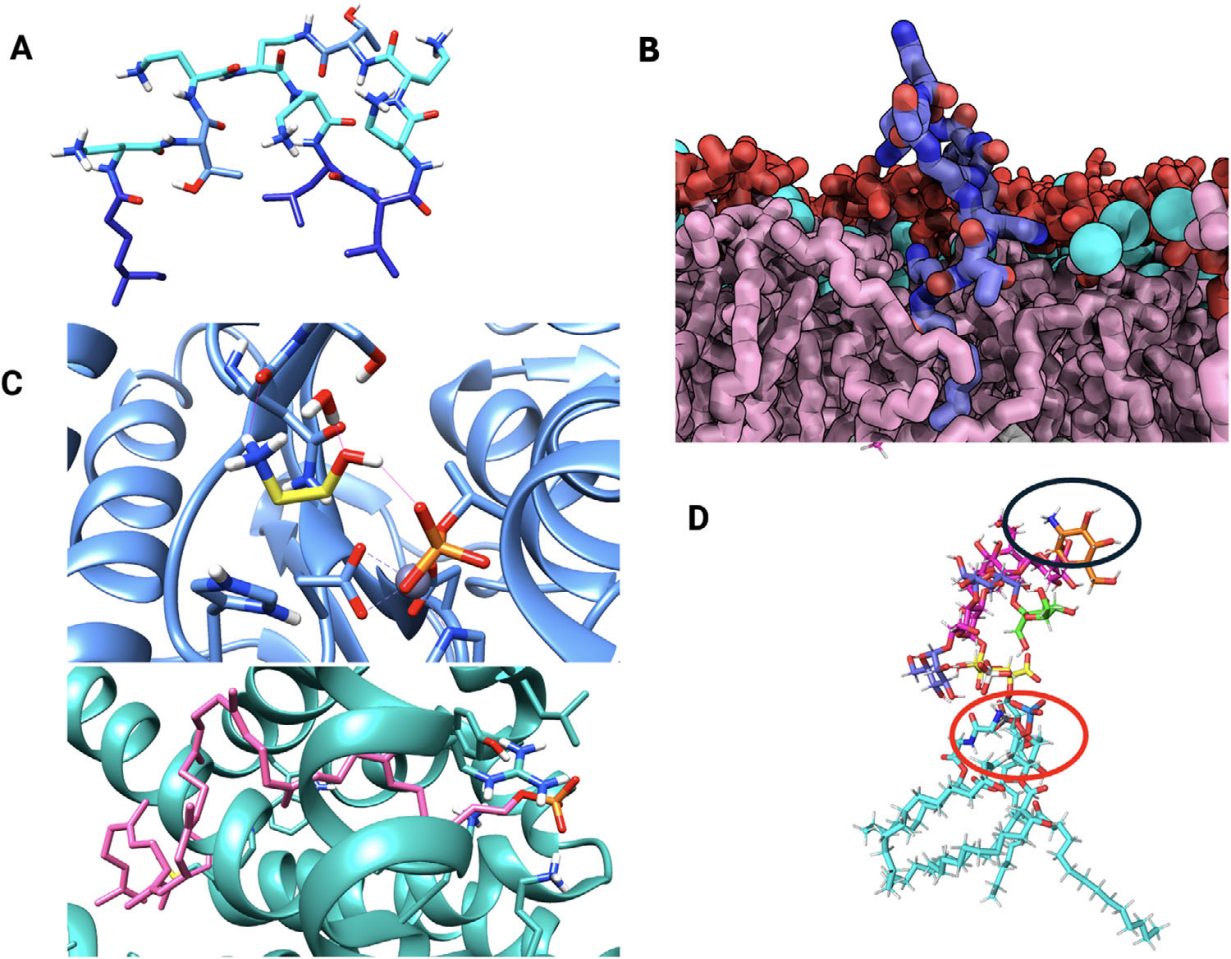
Structural insights into colistin and LPS modifications associated with colistin resistance. A) 3D structure of colistin (PDB ID: 8dev). Hydrophobic residues are shown in dark blue, polar residues in light blue, and cationic residues in cyan. B) Interaction model of polymyxin B with *E. coli* membrane with deep rough LPS. The lipid tail penetrates toward the hydrophobic core, while the cationic diaminobutyric acid residues interact with the membrane surface. Color scheme: lipid A in pink, Kdo sugars in red, polymyxin B in blue, and calcium ions in cyan. (C) Structures of resistance‐associated enzymes. The MCR‐1 complex (light blue) is shown with bound ethanolamine (yellow, PDB ID: 5yle), and ArnT (turquoise) is shown with a Lipid A precursor, undecaprenyl phosphate (hot pink, PDB ID: 5f15). MCR‐1 transfers ethanolamine to Lipid A phosphate groups, while ArnT adds 4‐amino‐4‐deoxy‐L‐arabinose. Both enzymes are involved in colistin resistance and hence possible drug targets for restoring antibiotic sensitivity. D) LPS structure of *V. cholerae El Tor*. The red circle highlights a di‐glycine modification; the black circle marks a glucosamine residue in the outer sugar core. Both features contribute to resistance against cationic AMPs such as colistin. Color scheme: lipid A in cyan, Kdo in yellow, heptose in magenta, fructose in green, glucose in blue, and glucosamine in orange.

The addition of 4‐amino‐4‐deoxy‐L‐arabinose (L‐Ara4N) by enzymes such as ColRS or PmrAB (**Figure** **11**) is another common modification conferring resistance to polymyxins.^[^ ^238^, ^279^, ^288^, ^296^, ^297^ ^]^ This bulky cationic sugar masks Lipid A phosphates (**Figure** **12**), thereby repelling cationic AMPs.^[^ ^238^ ^]^ Unlike pEtN,^[^ ^122^ ^]^ L‐Ara4N lacks Ca^2+^‐bridging properties but efficiently repels AMPs through charge shielding.^[^ ^122^ ^]^ In some cases, L‐Ara4N and pEtN coexist in the same LPS molecule, sometimes accompanied by additional palmitate acyl chains, creating LPS structures highly resistant to polymixins^[^ ^297^, ^298^ ^]^ via three synergistic mechanisms: enhanced LPS‐LPS interactions, charge repulsion, and improved hydrophobic packing.^[^ ^297^ ^]^ While L‐Ara4N is often attached to the Lipid A phosphates, in some species (e.g., *Proteus mirabilis*) it can also be attached to the Kdo residues of the core sugars.^[^ ^299^, ^300^, ^301^ ^]^ Additionally, hydroxylation of Lipid A acyl chains enables subsequent esterification reactions that incorporate new acyl chains.^[^ ^297^, ^302^ ^]^ This modification significantly increases membrane hydrophobicity and packing density, impeding the penetration of cationic AMPs. Notably, these more heavily acylated Lipid A variants are typically more immunogenic,^[^ ^289^, ^291^ ^]^ highlighting a trade‐off where increased AMP resistance is achieved at the expense of immune evasion.

*Vibrio cholerae* exhibits a peculiar modification of its acyl chains by attaching a glycine or di‐glycine substituent through an esterification reaction^[^ ^303^, ^304^ ^]^ (Figure 12D). This reaction involves the carboxyl group of the amino acid and a hydroxyl group on the acyl chain, leaving the amino group free and positively charged. Alongside this peculiar (di)glycine modification, *V. cholerae* employs other common LPS modification strategies, such as increasing the number of acyl chains and attaching pEtN moieties to Lipid A and core sugars.^[^ ^305^ ^]^ While the glycine modifications of acyl chains are a peculiarity of *V. cholerae*, the incorporation of glycine moieties into the core‐oligosaccharide of LPS is more widespread and observed in species such as *Haemophilus influenzae* and *Shigella flexneri*.^[^ ^306^ ^]^

*Shigella flexneri* also demonstrates a notable mechanism of resistance to cationic AMPs at the O‐antigen level. Typically, the O‐antigen repeats of *S. flexneri* consists of 4–5 sugar residues, three of which are L‐rhamnose. The serotypes Xv and Yv incorporate 1 and 2 pEtN substitutions, respectively, into the rhamnose residues of each O‐antigen repeat.^[^ ^307^ ^]^ These pEtN moieties strengthen bridging between O‐antigen repeat units, facilitated by divalent cations, forming an extensive interaction network that extends across 10–30 repeats.^[^ ^308^ ^]^ This dense barrier significantly impedes AMP penetration. Furthermore, these new *S. flexneri* serotypes evade immune recognition, presenting challenges for both antibiotic and vaccine development.^[^ ^307^, ^309^ ^]^

*Legionella pneumophila* is distinguished by an O‐antigen composition rich in Legionaminic acid,^[^ ^310^ ^]^ a nonulosonic sugar (nine‐carbon backbone). Although named after *L. pneumophila*, Legionaminic acid is also found in *Campylobacter* and *Acinetobacter* species.^[^ ^80^ ^]^ In *Acinetobacter*, Legionaminic acid has been linked to virulence and MDR.^[^ ^311^ ^]^ This sugar is critical for the integrity of LPS,^[^ ^310^ ^]^ but its direct contribution to AMP resistance remains unclear. Legionaminic acid effectively suppresses immune responses by mimicking eukaryotic sialic acid, a strategy employed by many bacterial species to avoid detection by the immune system.^[^ ^279^ ^]^

It is important to note that immune‐evading LPS modifications do not fully protect against endogenous AMPs, a component of innate immunity. Consequently, bacteria must evolve mechanisms to evade both the immune system and AMPs. For example, the LPS of *Helicobacter pylori* simultaneously exhibits AMP‐resistant and immune‐escaping features. Bacteria that cause chronic infections, such as *H. pylori*, are particularly adept at evading immune responses while withstanding the continuous presence of cationic AMPs in bodily fluids of mammals. This prolonged bacterial‐AMP co‐evolution has equipped bacteria with mechanisms to resist even last‐resort antibiotics like polymyxins.

On the positive side, the intricate co‐evolution of LPS and AMPs provides valuable insights into resistance mechanisms, offering potential strategies to target the wide array of LPS structures and modifications. Targeting LPS can involve designing novel LPS‐disrupting peptides or developing inhibitors for key LPS‐editing pathways, such as the catalytic pockets of Mcr‐1 and ArnT (**Figure** 12C).

### 3.3 Mammalian Cells

Eukaryotic cell membranes are characterized by a lipid bilayer structure primarily composed of phospholipids and cholesterol (structure model in Figure 7D, percentage composition in Figure 8). This section focuses on red blood cells (RBCs) as the primary mammalian cell model due to the abundance of lipidomics data and the routine use of hemolysis assays to estimate AMP toxicity. Notably, immune cells may exhibit significant differences in membrane composition^[^ ^312^, ^313^, ^314^ ^]^ and play critical roles in AMP activity and toxicity.^[^ ^249^, ^315^ ^]^ Nevertheless, mechanisms underlying AMP activity are less well‐studied for immune cells compared to RBCs.

The outer leaflet of the RBC plasma membrane is rich in polyunsaturated PC and saturated or monounsaturated SM.^[^ ^316^ ^]^ In contrast, the inner leaflet exhibits a nearly equal distribution of anionic lipids, such as PS and phosphatidylinositol (PI), alongside zwitterionic PC and PE.^[^ ^316^ ^]^ The inner leaflet's PC lipids are less unsaturated compared to those in the outer leaflet, while other lipids, including PE, PS, and PI, contain a higher proportion of polyunsaturated acyl chains. The outer leaflet's lipid acyl chains are more ordered and diffuse more slowly than those in the inner leaflet, probably resulting in greater stiffness in the outer membrane.^[^ ^316^ ^]^

This relative stiffness has critical implications for cellular processes, as membrane fusion^[^ ^317^, ^318^ ^]^ and pore formation^[^ ^54^, ^319^, ^320^, ^321^, ^322^ ^]^ depend on membrane curvature. The rigidity of the outer leaflet protects cells against sharp negative membrane curvatures induced by viral fusion proteins or endogenous AMPs.^[^ ^190^, ^323^, ^324^, ^325^ ^]^ This rigidity contrasts with the Gram‐negative IM,^[^ ^252^, ^283^, ^284^, ^326^ ^]^ where the outer leaflet is rich in curvature‐promoting PE.^[^ ^327^, ^328^ ^]^ PE plays a crucial role in AMP‐mediated pore formation,^[^ ^190^, ^329^ ^]^ and this difference in curvature behavior may be key to designing AMPs with greater selectivity.

A fundamental distinction between mammalian and bacterial membranes is the abundant presence of cholesterol in mammalian membranes, a molecule absent in bacterial membranes. Cholesterol imparts unique properties to eukaryotic membranes, including the formation of lateral domains often referred to as lipid rafts, which have critical biological functions.^[^ ^330^, ^331^, ^332^ ^]^ Cholesterol preferentially interacts with saturated lipids, promoting the formation of lipid rafts, particularly with sphingomyelin on the outer leaflet.^[^ ^333^, ^334^, ^335^, ^336^, ^337^ ^]^ These nano‐ or microdomains play significant roles in protein recruitment,^[^ ^337^, ^338^, ^339^, ^340^ ^]^ and the differences of the biophysical characteristics between raft and non‐raft domains probably affects AMP interactions.^[^ ^341^ ^]^ Interestingly, while increasing the energetic barrier for membrane permeation, cholesterol may in parallel keep the membrane highly elastic, a process termed diffusional softening.^[^ ^342^ ^]^

Cholesterol serves as an attractive “anti‐target” in the design of AMPs with enhanced selectivity toward bacterial over mammalian membranes. While the role of electrostatic interactions in AMP selectivity is well established — bacterial membranes are typically negatively charged due to components such as LPS in Gram‐negative and PG lipids in Gram‐positive bacteria^[^ ^341^ ^]^ — the influence of cholesterol is less clearly defined. Cholesterol is absent in most bacterial membranes but abundant in mammalian ones, where it modulates membrane fluidity, lipid packing, and domain organization. Cholesterol may impair AMP activity by promoting lipid order, reducing membrane permeability, or altering lateral pressure profiles. These biophysical effects likely contribute to the reduced susceptibility of eukaryotic membranes to AMP‐induced disruption. The contributions of electrostatic and steric factors to AMP selectivity, and their implications for rational peptide design, are discussed in detail in Section 4.2.

### 3.4 Concluding Remarks of the Membrane Section

To accurately reflect the diversity among bacterial strains and species, it is crucial to develop more sophisticated bacterial membrane models, informed by lipidomics studies. Incorporating cyclopropane‐containing acyl chains, branched acyl chains, lysyl lipids, and glycolipids in varying proportions enables the modeling of different bacterial species and strains, distinct phases of the cell cycle, and the states associated with AMP susceptibility or resistance. Similarly, the extraordinary variability of LPS, with the numerous resistance‐inducing modifications, must be captured to study their role in adaptive mechanisms. These enhanced models would also facilitate investigations into membrane domains, which play critical roles in various cellular processes,^[^ ^255^, ^343^, ^344^ ^]^ including AMP‐induced domain formation as a mechanism of membrane disruption.^[^ ^50^ ^]^

Lateral heterogeneities in lipid organization such as the aforementioned formation of lateral domains or “lipid rafts” have for long been proposed to play a role in bacterial functioning and especially extracellular communication.^[^ ^345^ ^]^ For bacteria, one here especially has to consider the inner and outer membranes and also the very strong asymmetric distributions of lipids and proteins across the membrane leaflets as well as along different regions of the usually polarized bacteria such as between polar and septal regions. This all is inter‐connected to highly curved membrane regions, all in all influencing also action of AMPs.^[^ ^346^ ^]^ Whether changes in membrane curvature or lipid ordering of fluidity, all these properties can in principle be studied with microscopic techniques such as fluorescence microscopy (using for example environment‐sensitive dyes reporting on local lipid membrane order or techniques such as fluorescence correlation spectroscopy measuring local molecular mobility and thus fluidity) or AFM (observing local membrane topology or stiffness).^[^ ^347^ ^]^ Here, bacterial membranes are difficult to investigate with these tools due to their more complex membrane structure compared to mammalian cells as well as increased difficulty to introduce membrane labels. However, recent data has highlighted progress in this respect.^[^ ^348^ ^]^ Another issue is that experiments on live bacteria usually entail laboratory environments with high biosafety levels, which are less equipped with high‐end observation tools such as advanced microscopes. Here, bacterial model membrane systems such as supported lipid membranes or giant unilamellar vesicles mimicking bacterial membranes through certain instituted lipid and protein mixtures come into play, which also allow to investigate membrane lateral organization and curvature in a more controlled way (e.g., by controlling lipid compositions). While these systems are good models for understanding basic physicochemical properties and functions, they are quite far away from the real complexity of bacterial membranes. For this, research has started to employ membranes or vesicles isolated from bacteria and e.g., examine the influence of external stressors on their organization.^[^ ^349^ ^]^ Also, Outer Membrane Vesicles (OMVs) extracted from the outer membrane of bacteria have shown reactions to antibacterial agents, depicting the living cells in a good manner.^[^ ^350^ ^]^

For eukaryotic membranes, insights into the structural complexity and the formation of raft domains are essential for understanding mechanisms underlying AMP selectivity. The lateral heterogeneity of mammalian cell membranes is indispensable for the hemolytic activity of certain peptides,^[^ ^351^, ^352^, ^353^ ^]^ emphasizing that simple homogeneous membrane models cannot adequately capture the nuanced mechanisms underlying AMP toxicity. Therefore, incorporating more realistic and complex membrane models in experiment and simulation, reflective of both bacterial and eukaryotic systems, is critical to advancing our understanding of AMP activity and selectivity.

## 4 AMPs Design and Discovery: From Natural Peptides to Therapeutics

This section explores the strategies and methodologies employed in the design and discovery of novel AMPs, transitioning from natural templates to therapeutic candidates. The process can be broadly divided into two phases. The first phase, *hit generation*, focuses on leveraging existing structures and learning from natural and synthetic AMPs to generate novel candidates with potential antimicrobial activity. The second phase, *hit‐to‐lead optimization*, delves into refining these candidates to enhance selectivity and activity while minimizing toxicity. Structural models and advanced computational approaches play a pivotal role in navigating the delicate balance between efficacy and safety, providing a foundation for rational AMP design and development.

### 4.1 The Hit Generation Phase: Finding a Needle in a Haystack

Advances in artificial intelligence (AI), ML, and bioinformatics have transformed AMP discovery by leveraging patterns from diverse methods or datasets to generate novel peptide sequences. These methods integrate data from MD, fluorescence imaging, and experimental assays, creating efficient discovery pipelines.

Das et al.^[^ ^31^ ^]^ demonstrated the power of such an approach, employing deep learning and unlabeled data alongside known AMPs to teach a model the “peptide grammar.” This method generated novel sequences, with the best candidates screened through CG‐MD simulations using the Martini force field^[^ ^19^ ^]^ and *insane*.^[^ ^354^ ^]^ The top 20 peptides from this shortlist were tested in vitro, showing the CG‐MD model predicted antimicrobial activity with an 88% sensitivity and 63% specificity. While CG‐MD of simplified model systems excels in high‐throughput screening, all‐atom MD simulations are essential for detailed mechanism‐of‐action studies.

Learning peptide grammar was also emphasized by Nagarajan et al.,^[^ ^355^ ^]^ who used a long short‐term memory (LSTM) language model trained on MIC data, instead of using a binary description of activity. Two out of the top ten resulting peptides showed high activity against multidrug‐resistant (MDR) Gram‐negative strains and reasonable toxicity, despite the absence of toxicity‐specific training data. This suggests inherent selectivity encoded in natural AMP sequences.

Incorporating non‐canonical amino acids (ncAAs) can expand AMP diversity beyond the “AMP‐similarity basin” resulting from many ML models. Maccari et al.^[^ ^356^ ^]^ *de‐novo* designed AMPs using a Maximum of Auto‐ and Cross‐Covariances (MACC) algorithm, reporting two peptides with broad‐spectrum activity. The same algorithm was also used to optimize a non‐active starting structure into an active AMP using an extended amino acid alphabet of 87 residues. ncAAs like those found in lugdunin (thiazolidine)^[^ ^110^ ^]^ or QPX‐9003 (chlorobenzyl moiety)^[^ ^128^ ^]^ exemplify how structural novelty may enhance AMP activity.

Designing AMPs for specific tasks rather than general antimicrobial activity is another promising direction. Lee et al.^[^ ^52^, ^323^ ^]^ trained SVMs to predict the ability of linear and cyclic peptides to induce negative Gaussian curvature (NGC) in membranes, a critical step for membrane disruption.^[^ ^190^, ^324^, ^357^ ^]^ Differentiating between AMPs and Cell Penetrating Peptides (CPPs) often relies on pore size, with AMPs forming larger pores and CPPs smaller ones.^[^ ^52^, ^217^, ^357^, ^358^, ^359^, ^360^, ^361^, ^362^, ^363^, ^364^ ^]^ However, computationally distinguishing these classes is challenging due to overlapping features like hydrophobicity and cationicity. Many peptides exhibit dual functionality, with CPPs displaying membrane‐disrupting activity at higher concentrations.^[^ ^365^ ^]^ The SVM model by Lee et al.^[^ ^323^ ^]^ effectively captured the lower hydrophobicity characteristic of CPPs compared to AMPs. Higher hydrophobicity in AMPs likely extends their membrane residence time, enabling the formation of stable, larger pores. In contrast, CPPs have shorter membrane residence times and smaller pore sizes.^[^ ^52^, ^313^ ^]^ Notably, CPPs often feature multiple arginine residues, which are more effective at inducing membrane translocation compared to shorter cationic side chains such as lysine, ornithine or 2,4‐diaminobutryic acid,^[^ ^216^, ^217^, ^366^ ^]^ frequently found in AMPs.

Fjell et al.^[^ ^367^ ^]^ utilized a “needle in a haystack” strategy, employing HMMs and cheminformatic techniques based on inductive QSAR descriptors^[^ ^368^ ^]^ to predict AMP sequences from unidentified proteins. This approach achieved a remarkably high hit rate in identifying active AMP sequences. Notably, the choice of input conformation — whether non‐minimized, gas‐phase minimized, or minimized in a Born implicit solvent — did not significantly impact model accuracy or sensitivity.^[^ ^369^ ^]^ This suggests that complex conformational effects are negligible for short, unstructured peptides. The virtual peptide library generated using this cheminformatic approach exhibited high frequencies of tryptophan, arginine, and lysine.^[^ ^369^ ^]^ Tryptophan, in particular, was enhanced in the most active quartile, underscoring its critical role in promoting membrane insertion. In contrast, physicochemical properties such as charge, hydrophobicity, and amphipathicity were similar across all quartiles, from the most to the least active peptides. This indicates that the relative positioning of amino acids within the sequence is a key determinant of antimicrobial activity.^[^ ^369^ ^]^

Reliable and curated experimental data form the foundation of AMP prediction models. Thomas et al.^[^ ^370^ ^]^ significantly advanced this field by creating and curating the CAMP (Collection of Anti‐Microbial Peptides) database. This resource was used to train three machine learning models — Discriminant Analysis (DA), RF, and SVM — to predict antimicrobial activity.^[^ ^370^ ^]^ Both RF and SVM demonstrated high performance with AUC values exceeding 0.8, whereas DA achieved a lower AUC of 0.74.^[^ ^370^ ^]^ The relatively poor performance of DA likely stems from the assumption of Gaussian distribution of the underlying variables, which are inadequate for capturing the non‐linear relationships typical of AMP properties and activity. SVM models, in particular, excel at AMP prediction due to their ability to effectively handle small datasets — often limited to hundreds or a few thousand sequences for AMPs — while managing high‐dimensional data and non‐linear relationships through diverse kernel functions. This robustness makes SVMs especially suited for the complex and multi‐faceted nature of AMP activity prediction.

#### 4.1.1 Lessons Learned From AI and Bioinformatic Approaches

The sequence‐based design of AMPs benefits from both simpler models and advanced algorithms, yet their outputs often converge on well‐established determinants of membrane activity, such as cationicity and hydrophobicity.^[^ ^371^ ^]^ While simpler models often arrive at peptide sequences similar to already known AMPs, global approaches like the one of Das et al.^[^ ^31^ ^]^ demonstrate greater novelty by leveraging large unlabeled datasets. Effective AMP design often pairs basic physicochemical properties (e.g., hydrophobicity, net charge) with structural motifs and positional information. For instance, Fjell et al. achieved strong performance using computationally derived QSAR descriptors,^[^ ^369^, ^372^ ^]^ with model accuracy largely unaffected by input 3D structures.

For short peptides (8–12 residues), positional information, such as the role of terminal residues and modifications, is critical for stability and activity.^[^ ^71^, ^290^, ^371^, ^373^ ^]^ These peptides are often unstructured in solution, making precise knowledge of the peptide fold less relevant. In contrast, longer peptides rely on secondary structure annotations, as folding becomes crucial. Protein‐specific^[^ ^374^ ^]^ and peptide‐specific^[^ ^85^ ^]^ structure prediction tools are poised to enhance the accuracy of folding predictions.

The multifunctionality of many AMPs^[^ ^161^, ^162^, ^375^, ^376^, ^377^ ^]^ complicates the identification of features driving individual mechanisms of action.^[^ ^323^ ^]^ While generative models consistently produce active and selective peptides, their outputs often remain within the existing AMP chemical space. The inclusion of non‐canonical amino acids could significantly diversify the peptide repertoire, but their low frequency and poorly understood behavior limit their integration into predictive models.

Cyclic peptides are less explored by ML models, and most studies are based on experimental medicinal chemistry and SAR analyses. Subtle chemical modifications often lead to significant activity changes,^[^ ^102^, ^113^, ^128^ ^]^ complicating activity prediction. While one ML model successfully classified NGC‐causing cyclic peptides and showed that the sequence design of linear and cyclic peptides are close cognates,^[^ ^52^ ^]^ this finding contrasts with the observation that linearized polymyxins lose activity,^[^ ^119^ ^]^ highlighting the unique properties of cyclic peptides.^[^ ^116^, ^378^, ^379^, ^380^ ^]^ Dedicated models for cyclic peptides are urgently needed to better harness their distinct features.

### 4.2 Hit‐to‐Lead Phase: Enhancing AMPs Selectivity

Having reviewed methods to generate novel AMPs, we now focus on improving their selectivity, a critical step for translating active peptides into viable drugs. During the hit‐to‐lead phase, MD simulations act as atomistic microscopes, enabling detailed studies of AMP‐membrane interactions and informing structureoptimization.

#### 4.2.1 Cholesterol and Selectivity

Cholesterol is a key component of human cell membranes and plays a crucial role in differentiating bacterial and human cell membranes. Its presence, particularly alongside polyunsaturated fatty acids (PUFAs), increases membrane mobility, softens the membrane, and induces lipid condensing and thickening.^[^ ^342^, ^381^, ^382^, ^383^ ^]^ Noteworthy, RBC membranes, the most studied for membrane composition and AMP toxicity, are indeed rich in PUFAs and cholesterol.^[^ ^316^, ^384^ ^]^

Cholesterol contributes to membrane functionality by maintaining membrane plasticity, while simultaneously impeding the permeation of xenobiotics and ions.^[^ ^342^, ^382^ ^]^ Its role in reducing membrane water permeability^[^ ^385^, ^386^, ^387^ ^]^ suggests that AMPs may face challenges in transporting water molecules during the initial pore‐formation steps. Additionally, cholesterol promotes lateral membrane heterogeneity, leading to distinct membrane domains with varying susceptibilities to AMPs. These phenomena — membrane thickening, lipid condensing, reduced water permeability, and increased lateral heterogeneity — highlight cholesterol's particular role and provide key insights for designing potentially more selective peptides.

#### 4.2.2 How Cholesterol Affects Peptide Insertion: Experimental and Computational Evidence

Understanding how cholesterol influences peptide insertion, using experimental and computational techniques, reveals critical mechanisms behind AMP selectivity. Ramamoorthy et al. and Hallock et al. studied Pardaxin, a peptide with strong bacteriolytic but weak hemolytic activity, using solid‐state NMR.^[^ ^319^, ^388^ ^]^ Pardaxin's helix‐hinge‐helix structure^[^ ^389^, ^390^ ^]^ enables membrane destabilization and possibly formation of a toroidal pore.^[^ ^69^, ^319^, ^388^ ^]^ However, cholesterol‐rich domains resist Pardaxin insertion by increasing acyl chain order and membrane thickness,^[^ ^342^ ^]^ limiting the hydrophobic matching required for pore formation.^[^ ^388^ ^]^ Cholesterol also inhibits the membrane curvature necessary for pore formation.^[^ ^54^, ^319^ ^]^ In the CG‐MD Martini simulations performed by Su et al.^[^ ^391^ ^]^, a DPPC/DLiPC/Cholesterol membrane was used as a model for a strongly phase‐separating membrane, along with the four peptides Magainin 2, BP100, MSI‐78, and MSI‐103. The study observed a depletion of all peptides from the liquid‐ordered phase (comprised of DPPC and cholesterol). This phenomenon was attributed to the more favorable lipid‐lipid interactions in the liquid‐ordered phase in the absence of peptides that compensate for the entropically unfavorable sorting of the peptides. Interestingly, the simulation also revealed that the peptide Magainin 2 formed toroidal pores only in the liquid‐disordered region of the membrane.

The selective pressure for reduced self‐damage in higher species may explain why endogenous AMPs show some degree of cholesterol‐induced selectivity.^[^ ^55^, ^362^, ^392^, ^393^, ^394^ ^]^ Sood et al. found that cholesterol, more effectively than lanosterol or ergosterol, prevents insertion of the toroidal‐pore forming peptides LL‐37(F27W) and Temporin L,^[^ ^395^ ^]^ likely due to its enhanced raft‐forming,^[^ ^396^, ^397^, ^398^ ^]^ lipid‐condensing, and membrane ordering abilities.^[^ ^330^, ^399^ ^]^ However, the simple SOPC/Chol models used in these studies overlook lateral heterogeneity, a key factor of the effect of cholesterol on biomembranes.^[^ ^400^ ^]^

For detergent‐like peptides such as δ‐lysin, Pokorny et al. demonstrated that cholesterol‐rich raft domains resist peptide activity, while liquid‐disordered POPC‐rich domains are vulnerable to attack.^[^ ^351^ ^]^ Paradoxically, cholesterol abundance can enhance hemolytic activity by creating smaller, disordered domains where peptides preferentially aggregate.^[^ ^351^ ^]^

Cholesterol is capable of inducing some degree of resistance against membrane‐active AMPs but becomes less effective in laterally heterogeneous membranes.^[^ ^353^ ^]^ Liquid‐disordered domains are especially susceptible to AMPs.^[^ ^341^, ^351^, ^352^, ^353^ ^]^ The literature consistently shows that toroidal pore‐forming α‐helical peptides are hindered by cholesterol‐induced acyl chain ordering and lipid compression. In contrast, cholesterol's role in detergent‐like peptide activity appears more variable, depending on its local concentration and the degree of lateral heterogeneity.

Cholesterol‐induced selectivity has been observed in cyclic peptides such as Lugdunin,^[^ ^110^ ^]^ S‐thanatin,^[^ ^401^ ^]^ and Gramicidin S.^[^ ^402^, ^403^ ^]^ These peptides exhibit widely varying physicochemical properties: Lugdunin is lipophilic and neutral, Gramicidin S is amphipathic, and S‐thanatin is polar and highly cationic. Also their pore‐forming mechanisms differ significantly: Lugdunin forms tubes,^[^ ^110^ ^]^ Gramicidin S forms cation‐specific channels after inducing membrane demixing and thinning,^[^ ^404^, ^405^, ^406^ ^]^ and S‐thanatin destabilizes LPS by displacing divalent cations.^[^ ^407^, ^408^ ^]^

The diversity in structure and function among these peptides makes the identification of a unifying principle for their cholesterol‐induced selectivity challenging. However, the lipid‐condensing effect of cholesterol^[^ ^330^, ^342^ ^]^ is a common explanation, as cyclic AMPs often show shallower membrane penetration in cholesterol‐rich environments.^[^ ^110^, ^401^ ^]^ The cyclic structure also enables tighter control of conformational space, facilitating targeted modifications to enhance selectivity and reduce hemolysis, as demonstrated with Gramicidin S derivatives.^[^ ^409^ ^]^

MD simulations, paired with experimental methods, offer valuable insights into the cholesterol‐induced selectivity of certain AMPs and the promiscuous behavior of others, such as Melittin.^[^ ^410^ ^]^ The study of cholesterol‐containing membranes is essential because the differential insertion of AMPs into bacterial versus RBC membranes is influenced by the physical properties of the membrane.^[^ ^54^ ^]^ Cholesterol can overshadow electrostatic interactions by protecting even anionic bilayers, typical targets of cationic peptides, from antimicrobial activity.^[^ ^411^ ^]^ Therefore, examining peptide interactions solely with zwitterionic or anionic membrane models fails to capture the complexity underlying peptide selectivity.

Realistic membrane models are crucial to account for eukaryotic membrane properties. For instance, the low bending modulus of RBC membranes (4 − 6*k*_*B*_ *T*) compared to 10*k*_*B*_ *T* for a POPC:Cholesterol model membrane (ration 6:4)^[^ ^342^, ^412^, ^413^ ^]^ illustrates that incorporating cholesterol is a starting point but insufficient to replicate RBC properties.

As highlighted before, membrane properties such as formation of lateral domains or curvature, which all usually involve changes in cholesterol content, can well be studies with advanced microscopic techniques such as fluorescence, Raman or AFM, reporting e.g. on local membrane composition, fluidity and order, or curvatures. Recent advances in spatial and temporal resolution of these techniques have brought them closer to studying the molecular scale and sub‐second and ‐millisecond fast processes, i.e. reaching the scales accessible by MD simulations. In this way, MD simulations can be fed as well as tested by information from experimental data, and experiments can be guided by insights from MD simulations.^[^ ^414^ ^]^ This of course accounts also for studying the molecular details of the interaction of AMPs with bacterial membranes, potentially opening new routes to the design of improved antimicrobial actions.

The presence of cholesterol in eukaryotic membranes offers a valuable avenue for enhancing AMP selectivity and minimizing cytotoxicity. Curvature‐dependent mechanisms like toroidal pore formation are inhibited by cholesterol‐induced membrane stiffness of the outer membrane leaflet. Similarly, the formation of membrane nanotubes — an energetically demanding process — is hindered in cholesterol‐rich membranes due to their reduced deformability.^[^ ^404^, ^405^ ^]^ In contrast, carpet‐like mechanisms are often associated with hemolytic activity. For instance, cationic peptides like SAAP‐148, which preferentially bind to anionic phospholipids, show limited interaction with the zwitterionic membranes of RBCs, thus displaying reduced hemolysis. Conversely, peptides such as δ‐lysin, which rely heavily on hydrophobic interactions and target zwitterionic lipids, exhibit higher hemolytic potential.

Optimizing the physicochemical properties of AMPs — particularly hydrophobicity and charge — is therefore essential to maximize bacterial targeting while minimizing off‐target effects. Excessive hydrophobicity, although beneficial for membrane insertion, can lead to nonspecific interactions with eukaryotic membranes.^[^ ^415^ ^]^ Additionally, the high cationic character of AMPs enhances selectivity by exploiting the large negative transmembrane potential of bacterial membranes (typically ‐140 to ‐220 mV) compared to the much lower potential in RBCs (around ‐12 mV).^[^ ^416^, ^417^ ^]^

Ultimately, balancing charge and hydrophobicity is critical for designing AMPs with high antimicrobial potency and low cytotoxicity. These aspects are critical for advancing AMP design and will be further explored in the next section.

#### 4.2.3 Physicochemical Properties and Aggregation: Implications for Activity and Toxicity

The physicochemical properties of AMPs are highly variable, and their ability to form dimers, oligomers, and larger aggregates adds complexity. These supramolecular structures can exhibit properties distinct from their monomeric forms, influencing both activity and toxicity.

Initial peptide‐membrane recognition is often driven by electrostatic interactions. For many peptides, this is merely the first step, while others rely entirely on surface interactions to drive activity, such as peptides that promote the formation of anionic lipid domain formation.^[^ ^50^ ^]^ Hydrophobicity is another important determinant for membrane interaction and is pivotal in balancing activity with toxicity. For example, reducing the hydrophobicity of peptides like Colistin,^[^ ^128^ ^]^ LL‐37,^[^ ^418^ ^]^ and Gramicidin S^[^ ^409^ ^]^ has yielded derivatives with improved therapeutic indices, supporting evidence that promiscuous insertion into zwitterionic membranes is largely hydrophobicity‐driven.^[^ ^70^, ^71^ ^]^ Hydrophobicity refers not only to the content of hydrophobic amino acids but also their spatial organization: large uninterrupted hydrophobic surfaces proper of highly amphipathic peptides (with large hydrophobic moments) are correlated with a more stable α‐helical structure when interacting with zwitterionic and RBC‐like membranes and consequently higher toxicity.^[^ ^419^, ^420^, ^421^ ^]^ Reports of reduced hemolysis upon introduction of α‐helix destabilizing residues, like D‐enantiomers,^[^ ^419^, ^422^ ^]^ confirm the theory that helicity and amphipathicity need to be balanced to achieve selective membrane activity.^[^ ^49^, ^423^, ^424^ ^]^

C‐terminal amidation is a common modification that significantly influences antimicrobial and hemolytic activity by altering net charge and hydrophobicity.^[^ ^71^ ^]^ However, increased hydrophobicity from amidation can promote aggregation, potentially enhancing hemolytic activity.^[^ ^71^ ^]^ While large peptide aggregates often exhibit hemolytic properties, dimerization appears to promote antimicrobial activity.^[^ ^93^, ^425^, ^426^ ^]^ Aggregation at low peptide concentrations is frequently driven by hydrophobic interactions,^[^ ^71^ ^]^ but no universal causal relationship between aggregation and hemolysis has been established.^[^ ^427^, ^428^, ^429^ ^]^ In some cases, aggregation even reduces toxicity when peptide‐peptide interactions shield hydrophobic domains, preventing membrane access.^[^ ^430^, ^431^ ^]^

The membrane interactions of peptide aggregates — whether homo‐ or heterodimers, oligomers, or larger assemblies — can differ substantially from that of monomers. Their relative toxicity is highly case‐specific. The possible missing causality between aggregation and hemolysis is that promiscuous membrane activity is hydrophobicity‐driven, hence even non‐aggregating highly hydrophobic peptides are more likely to be toxic.

Peptide aggregation presents both challenges and opportunities in AMP design. Modulating aggregation properties may enhance activity against target membranes or mitigate toxicity to human cells. Moreover, aggregation affects pharmacokinetic profiles, such as half‐life and organ distribution,^[^ ^432^ ^]^ underscoring its importance in overall peptide performance. Molecular aggregation can well be studied with different experimental tools, also for smaller peptide accumulates, e.g. using fluorescence tools such as fluorescence correlation spectroscopy^[^ ^433^ ^]^ or advances in scattering microscopy techniques such as the weighing of proteins and their aggregates using interferometric Scattering (iSCAT) microscopy.^[^ ^434^ ^]^

### 4.3 How MD Simulations can Integrate with Experimental Studies?

Deciphering the mode of action of AMPs requires integrating experimental and computational techniques. MD simulations have emerged as indispensable tools for providing atomistic‐level insights, offering a detailed and dynamic view of AMPs' mechanisms of action. These simulations allow to quantify changes in membrane fluidity (e.g., lipid diffusion and lipid nanodomain formation^[^ ^332^, ^435^, ^436^ ^]^), acyl chain order parameters, membrane thinning, and hydrogen bonding. Long, unbiased simulations may also fully reconstruct the mechanism of action for some peptides. Biased pulling simulations are used to quantitatively determine the energetic profile of membrane approach and permeation,^[^ ^437^ ^]^ allowing to analyze the partition coefficients across different lipid compositions.^[^ ^342^ ^]^ MD simulations are particularly impactful for studying the large class of membrane‐active AMPs, whose mechanisms demand dynamic structural modeling. Current trends highlight four areas for improvement to maximize their utility in AMP design:

- **Complex Membrane Models**: Expanding the variety of lipids in simulations will better capture the complexity of bacterial and mammalian membranes. Realistic heterogeneous membranes can form domains potentially relevant to AMP activity.
- **Realistic Bacterial Membrane Models**: Avoid inclusion of PC lipids in bacterial membrane models, as they are minor components originating mostly from scavenging pathways. More representative models should focus on PE/PG lipids and include components like CL, MK, or glycolipids for specific applications.
- **Asymmetric Bilayers**: Incorporating bilayer asymmetry reflects a key structural feature of bacterial membranes and likely influences peptide interactions and pore formation.^[^ ^438^ ^]^
- **Modeling Gram‐Negative Outer Membranes**: For broad‐spectrum AMPs, there is a noticeable gap in studies focusing on interactions with LPS, leaving many mechanisms of action unresolved. Modeling these interactions can also elucidate potential synergistic mechanisms between AMPs and other molecules.

## 5 Conclusion

The alarming rise of multidrug‐resistant (MDR) and pan‐drug‐resistant (PDR) bacterial strains necessitates the development of effective, resistance‐evading antibiotic therapies. (AMPs are re‐emerging as a promising antibiotic modality, capable of addressing these challenges in synergy with small‐molecule antibiotics.

Historically, AMPs were discovered in competitive ecological niches where their evolution was driven by the need for antimicrobial activity while promoting a long‐term ecological sustainability. Clinical AMPs such as Daptomycin, Colistin, and Vancomycin were identified from soil samples,^[^ ^5^, ^6^, ^439^ ^]^ while preclinical compounds like Micrococcin and Lugdunin were isolated from sewage and the human nose, respectively.^[^ ^109^, ^176^ ^]^ These peptides often exhibit poor PK properties, including low solubility and short half‐life, traits likely optimized for maintaining long‐term ecological balance by selectively targeting competitors without harming mutualistic species,^[^ ^440^ ^]^ as illustrated in **Figure** **14**.

**Figure 13:**
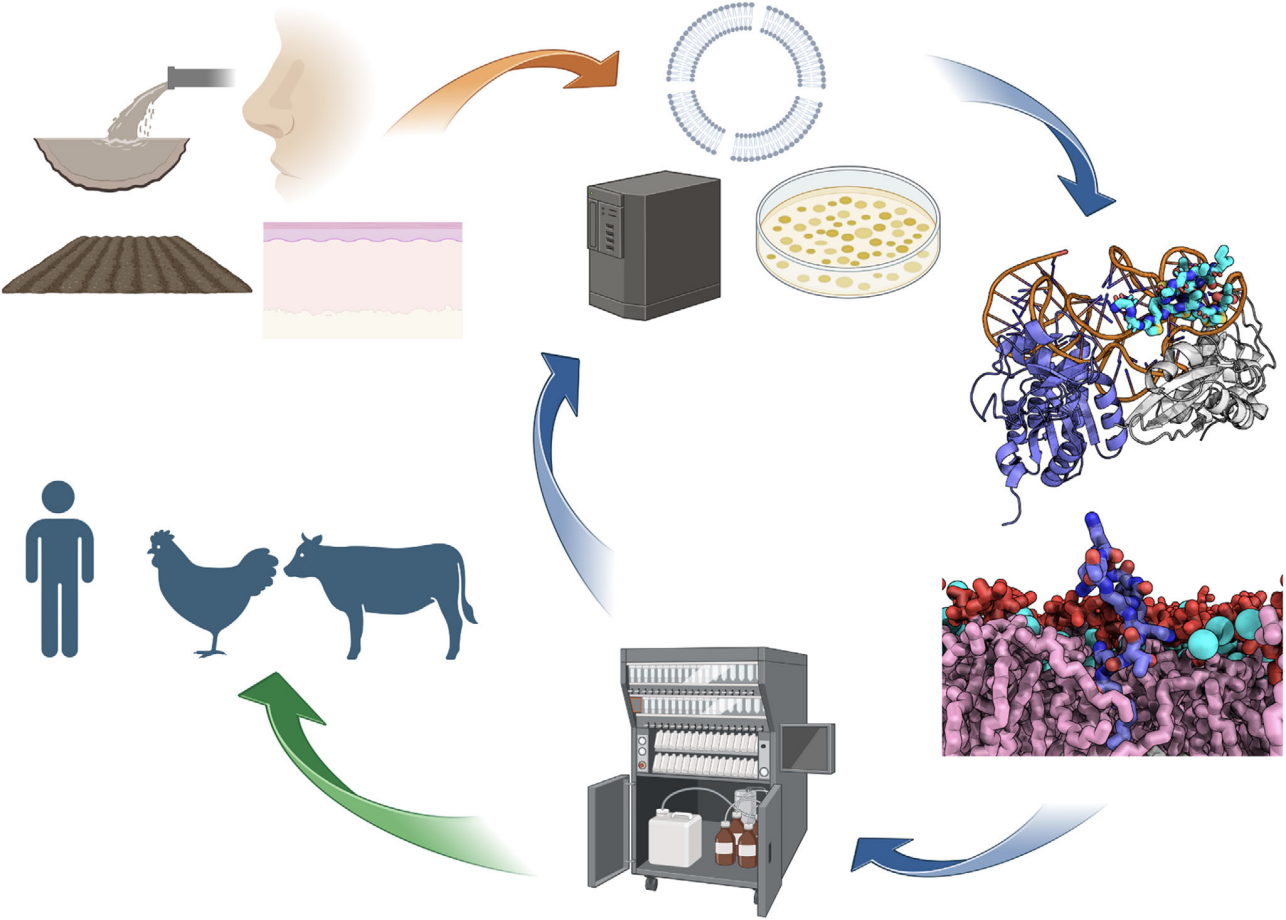
AMP Discovery: Integrating natural sources with experimental and computational approaches. Novel AMP sequences are often derived from microbial niches or endogenous peptides, yielding compounds with potent activity but varying stability and pharmacokinetic properties. These peptides are characterized using multiple methodologies to elucidate their mechanisms of action (MoA) at different scales, including atomistic modeling. Synthesis efforts are guided by these models, enabling a targeted search for effective compounds. AMPs are developed to address infections in both humans and animals, supporting a holistic approach to combating infectious diseases.

**Figure 14:**
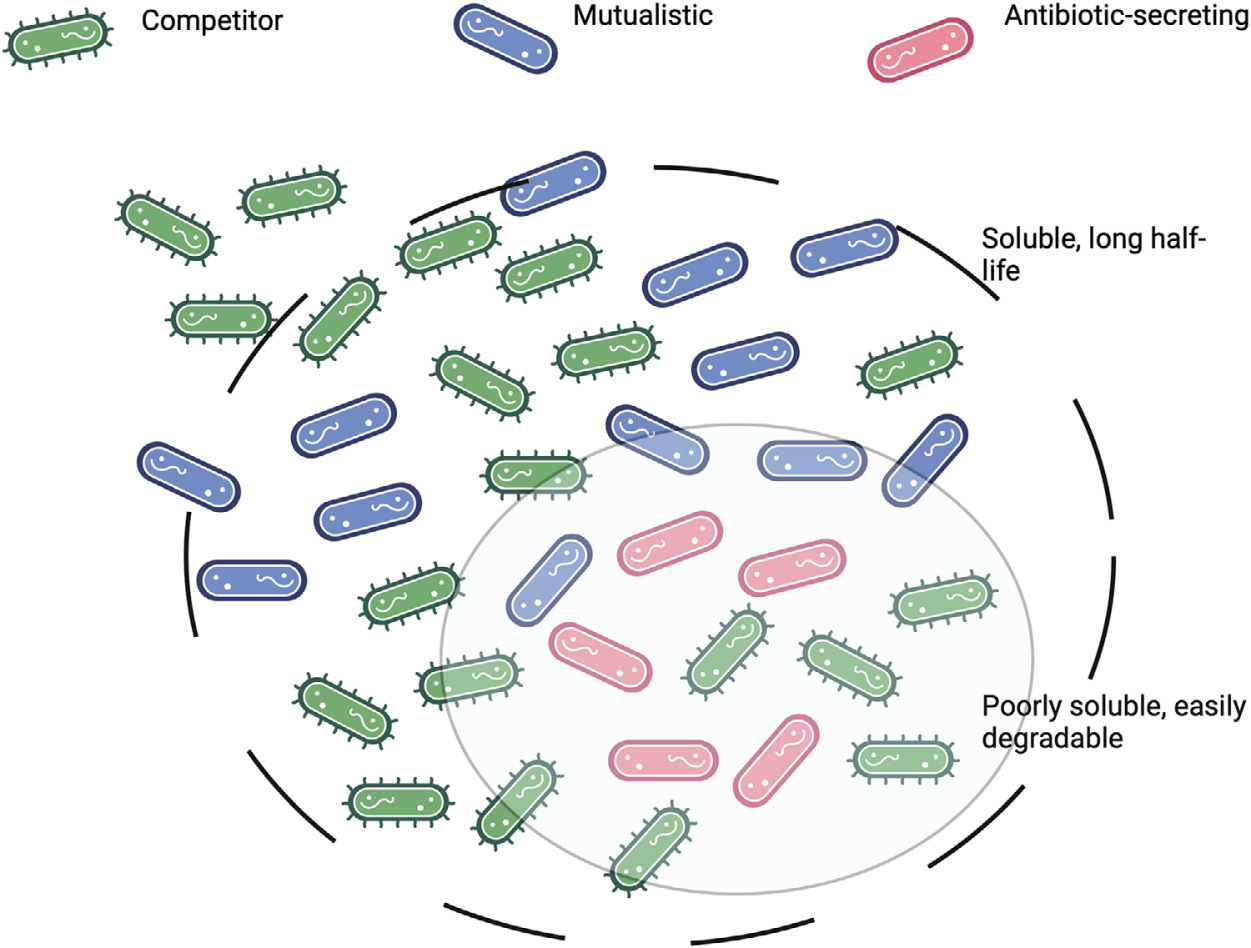
The solubility and half‐life of microbial AMPs may have evolved to be limited in order to reduce potential damage to their ecological niche. It is hypothesized that AMPs with prolonged half‐life and high solubility could overly disrupt the niche environment, which would be counterproductive for the antibiotic‐producing species.

PK limitations and safety concerns have historically hindered the clinical success of AMPs. Several AMP candidates, including pexiganan,^[^ ^441^ ^]^ surotomycin,^[^ ^442^ ^]^ and iseganan,^[^ ^443^ ^]^ failed to demonstrate superiority over standard treatments in clinical trials, resulting in their discontinuation. Others, such as CB‐182,804, murepavadin,^[^ ^444^ ^]^ and friulimicin B,^[^ ^445^ ^]^ encountered challenges due to safety or unfavorable PK profiles. Despite these setbacks, it remains difficult to draw definitive conclusions about the clinical viability of the AMP class, given the limited number of trials and the frequent lack of transparency regarding the reasons for candidate withdrawal.^[^ ^444^ ^]^

Nonetheless, the outlook for AMPs is shifting. Advances in peptide synthesis have made large‐scale production more accessible and cost‐effective. At the same time, the global rise of MDR infections has intensified the demand for new therapeutic modalities that target conserved bacterial structures. While classical antibiotics benefit from low cost and convenient oral administration, these same features have contributed to overuse and resistance development.^[^ ^446^ ^]^

Looking forward, clinical and regulatory benchmarks may evolve to include broader health impact metrics, such as effects on commensal microbiota,^[^ ^447^, ^448^, ^449^ ^]^ environmental persistence, and the potential to drive resistance. As technical and cultural barriers are overcome, AMPs are likely to play an increasingly prominent role in the future of infectious disease therapeutics.

Efforts to overcome the limitations of AMPs through structural fine‐tuning have demonstrated considerable success. Rational modifications, guided by structural models such as from MD simulations, docking studies, conformational sampling, X‐ray crystallography, and NMR spectroscopy, have led to the development of more effective peptide candidates.^[^ ^102^, ^103^, ^124^, ^128^, ^171^, ^175^, ^409^, ^450^ ^]^ In contrast, ML‐based approaches have so far struggled to produce fundamentally novel AMPs. This is largely due to the limitations of current models, which are typically trained on antimicrobial activity datasets — occasionally including hemolysis data — but often fail to incorporate the broader clinical challenges of AMP development, such as host cell permeability, hemolysis, serum protein binding, aggregation, and proteolytic stability.

At present, the rational optimization of existing scaffolds remains a more reliable path for generating clinically viable AMPs than *de novo* design. Addressing the intrinsic PK limitations of natural AMPs often requires specific structural modifications. Promising strategies include cyclization,^[^ ^451^ ^]^ stereochemical inversion,^[^ ^452^ ^]^ and the use of peptide‐like constructs.^[^ ^453^ ^]^ Furthermore, the same active pharmaceutical ingredient (API) can exhibit markedly different PK/PD depending on formulation,^[^ ^454^ ^]^ dosing regimen,^[^ ^455^ ^]^ or co‐administration with synergistic molecules,^[^ ^112^, ^456^ ^]^ providing additional avenues for optimization of AMPs clinical development.

The transition from active peptide hits to safe, effective therapeutics mirrors the broader success of peptide‐based drugs, where most approved agents are derived from endogenous hormones or other natural peptides through targeted modification strategies.^[^ ^377^, ^457^, ^458^ ^]^ As illustrated in **Figure** **13**, nature offers a diverse repertoire of bioactive compounds that serve as valuable templates for rational drug design. Mechanistic insights into peptide action are crucial for guiding these efforts, enabling fine‐tuning of molecular features such as stability, selectivity, and membrane interaction profiles.

This approach mirrors the successful “sharpening” of natural compounds into novel drugs, highlighting the potential of AMPs as a robust tool against the growing threat of antibiotic resistance. Here, further advancements in experimental observation technology will bring advancements, since they will lead to a close‐up of experiments and simulations, resulting in more sensitive and reliable data and predictions.

## Conflict of Interest

The authors declare no conflict of interest.
